# Management of eating disorders for people with higher weight: clinical practice guideline

**DOI:** 10.1186/s40337-022-00622-w

**Published:** 2022-08-18

**Authors:** Angelique F. Ralph, Leah Brennan, Sue Byrne, Belinda Caldwell, Jo Farmer, Laura M. Hart, Gabriella A. Heruc, Sarah Maguire, Milan K. Piya, Julia Quin, Sarah K. Trobe, Andrew Wallis, AJ Williams-Tchen, Phillipa Hay

**Affiliations:** 1 National Eating Disorders Collaboration, Sydney, Australia; 2 School of Psychology and Public Health, La Trobe University, Wodonga, Australia; 3 Department of Psychology, University of Western Australia, Perth, Australia; 4 Eating Disorders Victoria, Melbourne, Australia; 5 Lived Experience Advocate, Melbourne, Australia; 6 School of Psychology and Public Health, La Trobe University, Melbourne, Australia; 7 Eating Disorders and Nutrition Research Group (ENRG), School of Medicine, Western Sydney University, Sydney, Australia; 8 InsideOut Institute for Eating Disorders, The Boden Collaboration for Obesity, Nutrition, Exercise and Eating Disorders, The University of Sydney, Sydney, Australia; 9 Sydney Local Health District, NSW Health, Sydney, Australia; 10 School of Medicine, Western Sydney University, Macarthur Clinical School, Sydney, Australia; 11 Camden and Campbelltown Hospitals, Sydney, Australia; 12 Sydney Children’s Hospitals Network, The Children’s Hospital Westmead, Sydney, Australia; 13 Eating Disorders and Body Image (EDBI), Translational Health Research Institute, School of Medicine, Western Sydney University, Sydney, Australia; 14 South Western Sydney Local Health District, Sydney, Australia

**Keywords:** Guideline, Atypical anorexia nervosa, Bulimia nervosa, Binge-eating disorder, Other specified feeding or eating disorder, Obesity

## Abstract

**Introduction:**

The prevalence of eating disorders is high in people with higher weight. However, despite this, eating disorders experienced by people with higher weight have been consistently under-recognised and under-treated, and there is little to guide clinicians in the management of eating disorders in this population.

**Aim:**

The aim of this guideline is to synthesise the current best practice approaches to the management of eating disorders in people with higher weight and make evidence-based clinical practice recommendations.

**Methods:**

The National Eating Disorders Collaboration Steering Committee auspiced a Development Group for a Clinical Practice Guideline for the treatment of eating disorders for people with higher weight. The Development Group followed the ‘Guidelines for Guidelines’ process outlined by the National Health and Medical Research Council and aim to meet their Standards to be: 1. relevant and useful for decision making; 2. transparent; 3. overseen by a guideline development group; 4. identifying and managing conflicts of interest; 5. focused on health and related outcomes; 6. evidence informed; 7. making actionable recommendations; 8. up-to-date; and, 9. accessible. The development group included people with clinical and/or academic expertise and/or lived experience. The guideline has undergone extensive peer review and consultation over an 18-month period involving reviews by key stakeholders, including experts and organisations with clinical academic and/or lived experience.

**Recommendations:**

Twenty-one clinical recommendations are made and graded according to the National Health and Medical Research Council evidence levels. Strong recommendations were supported for psychological treatment as a first-line treatment approach adults (with bulimia nervosa or binge-eating disorder), adolescents and children. Clinical considerations such as weight stigma, interprofessional collaborative practice and cultural considerations are also discussed.

**Conclusions:**

This guideline will fill an important gap in the need to better understand and care for people experiencing eating disorders who also have higher weight. This guideline acknowledges deficits in knowledge and consequently the reliance on consensus and lower levels of evidence for many recommendations, and the need for research particularly evaluating weight-neutral and other more recent approaches in this field.

**Supplementary Information:**

The online version contains supplementary material available at 10.1186/s40337-022-00622-w.

## Plain English summary

The objective of this project was to develop recommendations and clinical considerations to guide clinicians in the management of people experiencing eating disorders who also have higher weight. A Guideline Development Group was formed containing members with academic and/or clinical expertise and people with a lived experience of eating disorder. The guideline was not only informed by reviews of the scientific literature but also clinical expertise and lived expertise. This guideline has undergone extensive review and consultation over an 18-month period involving reviews by key stakeholders, including experts and organisations with clinical, academic and/or lived expertise. The guideline outlines a set of recommendations for clinical practice including the strong recommendation for psychological treatment to be offered as the first treatment for an eating disorder in people who are of higher weight. Considerations in clinical practice including weight stigma, care by professionals from disparate disciplines, and cultural considerations are also discussed. The Guideline Development Group acknowledges a lack of available research evidence specific to people experiencing an eating disorder who are also of higher weight and consequently some recommendations relied on consensus of group members taking into account the expert reviews. The Group also identified areas where additional research is necessary such as research evaluating weigh-neutral and other more recent approached in the field.

**Supplementary Information:**

The online version contains supplementary material available at 10.1186/s40337-022-00622-w.

## Introduction

### Executive summary

Eating disorders are serious, complex and potentially life-threatening mental illnesses. While historically, eating disorders have been conceptualised as disorders of people of low body weight^1^ there is now substantive evidence that this is inaccurate. The most common eating disorders are binge-eating disorder, other specified feeding or eating disorder (OSFED) and bulimia nervosa, and these occur in people across a broad range of body types [1]. Eating disorders are common and increasing in prevalence. This is particularly true for people with eating disorders who are of higher weight. This population comprises more than half of all people with an eating disorder in Australia with rates of eating disorders increasing most in people with higher weight [2].

A key rationale for this guideline (see Box 1) is that despite the high prevalence, eating disorders in people with higher weight have been consistently under-recognised and under-treated. People with a lived experience of an eating disorder who are of higher weight report being misdiagnosed, dismissed by health professionals and sidelined or excluded from eating disorder treatment services. This population is also often absent from eating disorders research, with the exception of binge-eating disorder. Weight stigma is a major factor contributing to these shortfalls and is addressed in this guideline. This guideline aims to promote weight-inclusive practice and provide advice on how to avoid weight stigmatising practices for people with an eating disorder who are of higher weight.

**Box 1:** Lived experience perspectives: why we need this guideline

“In hindsight, I’ve lived with disordered eating since I was a child. It emerged following some traumatic experiences, but my eating disorder wasn’t first identified until I was 19 years old and presented at my General Practitioner [in the United Kingdom (UK)] for treatment for another mental illness. At that time, the eating disorder services in my area only accepted people with a low body mass index (BMI). Since moving to Australia at age 21, it's been a long journey of trying to access appropriate treatment, with periods where I've been well and managing, and several periods when I've relapsed. Currently, at age 32, I’d say I’m mostly well, but I still have periods where I binge, and others where I restrict ‘to compensate’, and food and eating remains something I’m hyper-vigilant about.
I feel very privileged to have been able to contribute to the development of this treatment guideline. For much of my recovery, I feel like I have been doing it alone, as I have struggled to access compassionate, equitable and effective treatment options for my eating disorder, as it presents in my higher weight body.
The most significant help I have received is access to high quality, trauma-informed psychological support. For so long, I believed—and others fed my belief—that my body was the way it was because of some inherent failure on my part. I’ve lost count of the number of times I’ve simply been told to ‘eat less, and exercise more’ (including by mental health professionals). That was never going to work for me—I needed psychological support that helped me to understand why I eat the way I do, and to provide me with other coping mechanisms when I need them.
Holistic care has also been central to this—health professionals across the range of services that I have accessed who understand my needs as a person with an eating disorder, whatever my weight. That means, a GP who understands that my eating disorder has prompted nutritional deficiencies, and treats them compassionately alongside my mental health needs, and a personal trainer who has taught me to love exercise for the way it makes me feel, not as a punishment.
But along the way, I have also experienced a lot of bad care. Health professionals who have made me feel ashamed for my weight and dismissed my concerns because I don’t look like the stereotypical eating disorder patient. They have frequently centred my weight and weight loss as the primary goal for my health—as though I wasn’t aware I was living at a higher weight! I’ve experienced a range of treatment from dismissive to outright discriminatory. Far from helping me recover, this care makes me feel worse about myself, usually leading to a worsening of my mental health (and often weight gain). I hope this guideline demonstrate the pernicious effects of weight stigma, right down to the language we use to describe eating disorders as they present in higher weight as somehow different from the same condition at a low weight (e.g., ‘atypical anorexia nervosa’). Everyone is deserving of equitable care, regardless of what their body looks like.
Earlier identification and access to support likely also would have helped my recovery. My eating was viewed primarily as a weight issue by me, my family and health professionals, which got me caught in a spiral of thinking I was never good enough. Support and education for family members would also have helped to communicate what was happening with me and why. There is still a perception that people with eating disorders are thin, young, white women. If you don’t fit into this body type, care can be dismissive, and there is an absence of culturally appropriate treatments and supports.
While I’ve experienced some very bad treatment, some has been good, too. This gives me hope that there’s a future for eating disorder treatment without the stigma. I hope too that this guideline showcase the positive and proactive approaches to the treatment and support that works.“
-*Jo Farmer, lived experience advocate and Guideline Development Group member.*
“I grew up chubby and as I entered adolescence and adulthood that became ‘overweight’ and then ‘obese’. I have a few physical health issues that caused me pain and discomfort and I was told that losing weight would help and I should eat smaller portions and try to exercise more.
The first time I got this advice I was 10 years old. At age 10, I was told by my doctor that no one would ever love me at my current weight and that following his advice would make me healthier and happier. I followed this advice but never lost weight, so I was repeatedly doubted by many different medical professionals, so they repeated their advice. I missed out on a lot of typical teenage experiences by being at the gym and avoiding situations where I’d have to eat with people.
My eating disorder wasn’t identified until I was admitted to an inpatient mental health unit at aged 20 but at the time, I wasn’t in a place to treat it as depression and anxiety took priority. It took a few years before I was ready to get treatment for it, and I addressed it with my psychologist of the time. I was told advice that over the course of my life I have become very familiar with, eat less and exercise more. If I lost weight, then there’d be less fuelling the issues behind my disordered eating. Or at least that’s how it was explained to me.
It took me some time to find a medical team I could feel comfortable with because even after explaining that I had an eating disorder, doctors would still tell me how much easier and better things would be if I lost weight. After 18 years and lots of support from family and medical professionals from various disciplines, I’ve stopped trying to lose weight and am focusing on healing my disordered relationship with food.
As I get older and my friends and family start having children, I’ve been worried that they will end up in a similar situation to what happened to me. My hope for these guidelines is that they teach people that eating disorders aren’t just for certain body types and that they lead to better supports in place for this under-recognised group, that the young people in my life can get diagnosis and appropriate treatment no matter what they look like.
*- Zoe Bower, lived experience advocate.*

The aim of this guideline is to synthesise the current best practice approaches to the management of eating disorders for people who are of higher weight, based on the premise that every person with an eating disorder is deserving of equitable, safe, accessible, and evidence-based care regardless of their body size. It accords with the role and function of the National Eating Disorders Collaboration (NEDC) to synthesise research evidence, clinical expertise and lived experience in national standards to improve systems of care for all Australians. While it is important to recognise eating disorders in people with ‘severe obesity’ or those presenting for bariatric surgery, it is important to note that the aim of this guideline is not to address weight loss or treatment of ‘obesity’.

In 2019, the NEDC Steering Committee auspiced this guideline and a Guideline Development Group was formed containing academic and/or clinical expertise, and/or lived experience from diverse disciplines. Modelled on the ‘Guidelines for Guidelines’ process outline by the National Health and Medical Research Council (NHMRC), the guideline was not only informed by recent systematic reviews, meta-analyses and primary trials, but also clinical expertise and lived expertise. It should also be noted that the voice of lived experience is largely absent in the literature. This guideline has undergone extensive peer review and consultation over an 18-month period involving reviews by key stakeholders, including experts and organisations with clinical and/or academic expertise and/or lived experience.

This guideline is intended for all health care professionals and does not present specialist information for any specific discipline. It does not aim to provide recommendations on prevention or detection but does provide advice on assessment. The guideline addresses treatment and/or management recommendations, specifically for people with an eating disorder who are of higher weight. This encompasses, but is not limited to psychological, pharmacological, nutritional, medical, family and activity interventions. Management should address all aspects of an eating disorder, thus interprofessional collaborative practice (ICP) is recommended, with each clinician practicing within the scope of their profession. Readers are referred to other literature for management of specific medical and other psychological disorders that are often experienced by people who have an eating disorder and are of higher weight.

It is hoped that this guideline will assist health care professionals in all relevant fields to understand the needs of people in their care who have an eating disorder who are of higher weight, and support the clinician in providing appropriate management of the eating disorder. Moreover, it is hoped that clinicians are more aware of, and responsive to, the adverse effects of weight stigma on the lives, health and treatment seeking of people with eating disorders who are of higher weight. This guideline acknowledge deficits in knowledge and consequently the reliance on consensus and lower levels of evidence for many recommendations, and the need for research particularly evaluating weight-neutral and other more recent approaches in this field.

### Scope

The aim of this guideline is to synthesise the current best practice approaches to management for people with an eating disorder who are at higher weight. The focus is on the treatment of the eating disorder (see “Background to eating disorders and how they occur” section for a definition of eating disorders), experienced in people living with higher body weight. The aim is not to address weight loss or “treatment of obesity”.

This guideline is intended for all health care professionals and does not present specialist information for any specific discipline. Where applicable, readers will be directed to resources for the latter. It is also not aiming to provide formal recommendations on prevention but does discuss clinical considerations of identification and assessment.

This guideline was developed within the Australian context and thus includes reference to Aboriginal and Torres Strait Islander peoples. However, it is anticipated to be relevant more widely as representing current knowledge and best health practice broadly. For this reason we have chosen to publish in international literature where it comes under scrutiny with international review. As the focus of this guideline is on the management of eating disorders, the outcomes considered are those relevant to the eating disorder. General physical and mental health-related quality of life are relevant as secondary outcomes. A reduction in body weight, or stabilisation of fluctuating body weight in itself is not an outcome or goal of treatment of an eating disorder experienced by people with higher weight. Further, it is possible that attempts at weight loss may exacerbate eating pathology and therefore may be contraindicated in some people (see Box 2).

**Box 2:** Weight loss and health in people with higher weight

It is common for people with an eating disorder to present seeking weight loss and to weight loss clinical programs [118]. The management of weight loss is outside the scope of this guideline and eating disorder providers should be cautious about engaging in weight loss advice. This guideline also acknowledges that this is an area of high contention in the field of eating disorders. Notwithstanding this caution, clinicians should be aware of the current evidence-based information that non-surgical weight loss is unlikely to be sustained in the longer-term [including behavioural weight loss interventions; 21, 22, 23] and impacts such as metabolic slowing, potential activation of a genetic predisposition to weight regain after weight loss, and the risk of relapse of the eating disorder. Whilst there is broad consensus that medically unsupervised weight loss regimes are likely to be unhelpful for people with eating disorders, no consensus was reached to make a recommendation in this guideline that a person with an eating disorder and higher weight should never attempt a medically supervised weight loss program. However, whilst there may be health benefits of moderate weight loss of 5–10% of body weight, clinicians should be aware of alternative approaches including non-diet weight-neutral approaches [e.g., HAES; 24, 25] with potentially similar health benefits such as improvements in lipid profiles and hypertension. Longer-term studies are needed of such weight-neutral approaches.
Eating disorder clinicians may advise and/or work in a multi-disciplinary metabolic, bariatric or similar medical clinic. This may be appropriate to support the essential need for screening, assessment and care of people with eating disorders presenting to such providers, and to increase awareness of weight stigma impacting on practice in such settings. In this context it is important to emphasise the importance of interprofessional collaborative practice (ICP; see “Management overview” section) and respecting the preferences of the person with the eating disorder and those who care for them. The presence of an eating disorder should not delay and does not preclude treatment for other medical/psychological conditions.

Notwithstanding that, we acknowledge that some people with significant medical co-occurring conditions or those presenting for bariatric surgery may require and seek significant weight loss, in the presence of diagnosed or undiagnosed eating disorders. We also acknowledge the complexities for people experiencing an eating disorder who are undergoing bariatric surgery and other weight loss regimes. While the management of obesity is not within the scope of this guideline, it is hoped that this guideline will assist health care professionals in all relevant fields to understand the needs of people in their care with an eating disorder, refer appropriately, and work collaboratively with other health professionals providing care and treatment for people experiencing eating disorders.

### Weight stigma

Weight stigma is the disparaging association of higher weight with negative personal characteristics [3]. ‘Weight stigma’ in this guideline is used to mean the occurrence of discrimination against or stereotyping of a person based on their weight, size or shape [4]. Other terms used are ‘sizeism’, ‘weight/size oppression’, ‘weightism’, ‘weight/size bias’, ‘weight-based discrimination’ and ‘fat phobia’.

Internalised weight stigma occurs when an individual upholds these disparaging associations towards their own body weight. Stronger internalised weight stigma predicts greater eating disorder psychopathology, higher levels of body dissatisfaction and poorer quality of life [5] and is common among people seeking bariatric surgery [6]. Stigma may also extend to the negative impacts of weight-stigma in parents of higher weight children [7].

Weight stigma has serious adverse impacts on the lives, health and treatment seeking of people with higher weight. Weight stigma may lead directly to disordered eating via complex neurobiological mechanisms, or with the aim of reducing the emotional distress it causes [8, 9]. There is active investigation into neurobiological mechanisms of weight stigma and the relationship with disordered eating [e.g., the research of 9–11]. Understanding and addressing weight stigma is crucial to the care of people with higher weight. Experiences of weight stigma, body shame or other negative emotions such as guilt are traumatic and may contribute to the onset of eating disorders and increase disordered eating in those with eating disorders [12–14]. Perceiving and experiencing a health care provider as weight stigmatising is associated with disengagement from treatment or health care [15, 16].

An important aspect in addressing weight stigma is in the use of language that avoids stigmatising terms for someone experiencing weight stigma. For this reason, this guideline use the phrases ‘people with higher weight’ and ‘living in a larger body’. Notwithstanding this approach, it is important to emphasise that there is not one universally preferred term for people living in larger bodies and health professionals should discuss preferred language with each person.

Despite being recognised for nearly half a century [17] weight stigma continues to be a major factor in the under-recognition and under-treatment of eating disorders, and especially of eating disorders experienced by people with higher weight. It is not well understood by the broader medical community that eating disorders among people of higher weight are just as serious and life threatening (from medical complications and self-harm) as eating disorders among people at lower weight. In addition, eating disorders at any weight are associated with a high level of psychological distress and psychopathology [18–20]. In reviewing the literature for this guideline, it is notable that the bias applies in both directions. That is, there are major gaps in the literature pertaining to both the treatment of binge-eating disorder (BED) for people at any size, and, more relevant to this guideline, the treatment of eating disorders other than BED in people at higher body mass indexes (BMIs).

Health professionals may be influenced by societal views on higher body mass and offer treatment tailored to a person’s weight rather than their eating disorder (e.g., advising a medication for its appetite suppressing effects rather than binge eating reduction). Health professionals need to be aware of the risks versus benefits of discussing body weight, particularly with people vulnerable to, or who have experienced an eating disorder. This guideline aims to promote weight-inclusive practice and advice on how to avoid weight stigmatising practices for people with an eating disorder who are of higher weight.

### Limitations of body mass index (BMI),^2^ language and definition of key terms

Cognisant of weight stigma and other considerations in this guideline, the terms *larger bodied* and *higher weight* includes people with high body mass index (BMI; kg/m^2^) through low adiposity and high muscle density (i.e., muscle building/athletes in larger bodies), as well as those with high adiposity. It may also include people with high adiposity but normal metabolic health indices and no physical heath co-occurring conditions [27] although these may develop in the future. Thus, this guideline does not define higher weight by a BMI cut off but rather focusses on a conceptualisation of a larger body that includes people who may be impacted socially and by the health system by standard BMI cut off points.

Historically BMI has been and continues to be widely used as an indicator of risk relating to physical health status. However, it is acknowledged that there are limitations to sole reliance on BMI [28]. As noted above, body composition can be highly variable in people with the same BMI and is influenced by many factors such as age, sex, race and muscularity. BMI has utility as a chronic disease risk marker in a population but should be used with other indicators of health status for a person. In individual assessment, other anthropometric, biochemical and behavioural measures may include waist circumference, blood pressure, blood glucose and lipid profiles. In children and adolescents, the height and weight growth velocity is preferred to the BMI. For all people it is more useful, if possible, to consider the person’s pre-illness growth trajectory as likely to be close to their ‘normal’ or ‘natural’ body habitus. This trajectory should be used to guide assessments of nutritional repletion and physical recovery. It is also important to note that people living in larger bodies, may have been engaged in weight suppression strategies for many years (in some instances, since childhood), and prior to the eating disorder, and thus their pre-illness BMI may yet be weight-suppressed rather than ‘natural’.

## Context

### Rationale for this guideline

Historically, eating disorders have been conceptualised as illnesses of people of low body weight [1] and typified by disorders such as anorexia nervosa. There is now substantive evidence that this is inaccurate. The most common eating disorders are BED, other specified feeding or eating disorder (OSFED)^3^ and bulimia nervosa, and these occur in people across a broad socio-demographic spectrum and a range of body types. This guideline address the particular issues that arise in the care of people experiencing eating disorders who are of higher weight. These individuals represent over half of all people experiencing an eating disorder in Australia with rates of eating disorders increasing most in people with higher weight [2]. The issues affecting people with eating disorders who are of higher weight are complex and important. These issues include delayed identification, misdiagnoses in assessment, subsequent inappropriate and inadequate treatment, widespread stigma, and the introduction of new disorders (i.e., anorexia nervosa without low weight). To our knowledge there are no current Australian guidelines to assist health professionals caring for people with both eating disorders and higher weight.

### Background to eating disorders and how they occur

The main DSM-5 eating disorders^4^ are described in Table 1. They comprise anorexia nervosa, bulimia nervosa, BED, avoidant restrictive intake disorder (ARFID), OSFED and unspecified feeding or eating disorder (UFED). Only one, anorexia nervosa, is defined by weight (i.e., underweight criteria). Where all features of anorexia nervosa are present except for low body weight, DSM-5 suggests a diagnosis of ‘atypical anorexia nervosa’. In most respects the World Health Organization ICD-11 [29] criteria closely match those of the DSM-5, though the ICD-11 does not require low-weight for a diagnosis of anorexia nervosa. For the purposes of this guideline, when providing advice on assessment or recommendations for treatment, the ICD-11 terminology for anorexia nervosa is adopted. That is, anorexia nervosa (code 6B80) is used as a broad term to include people at all body weights and without specifying the underweight criterion (sub coded in ICD-11 as 6B80.0, anorexia nervosa with significantly low body weight). The other eating disorders can occur in individuals across the weight spectrum.

**Table 1:** Overview of DSM-5 diagnostic criteria for eating disorders

	Anorexia nervosa	Bulimia nervosa	Binge-eating disorder	Avoidant/restrictive food intake disorder (ARFID)	Other specified feeding or eating disorder (OSFED)
Overvaluation weight &/or shape	Required	Required	May be present	Not present	May be present
Fear of fatness and/or behaviour preventing weight gain	Required	May be present	May be present	None but food is restricted	May be present
Underweight	Required^a^	Not present	Not present	May be present	May be present
Unmet nutritional and/or energy needs	Required	May be present	May be present	Required	May be present, likely in atypical anorexia nervosa
Weekly binge eating	May be present	Required	Required with distress and 3/5 descriptors^c^	Not present	May be present, likely in night eating syndrome
Weekly compensation^b^	May be present	Required	Not present	Not present	Likely in atypical anorexia nervosa and purging disorder but is not compensatory to binge eating
Remission specifier^d^	Partial/full	Partial/full	Partial/full	In remission	None
Severity specifier	BMI scale	Frequency of compensation	Frequency of binge eating	None	None

If anorexia nervosa is present, by definition no other eating disorder will be present. If avoidant/restrictive food intake disorder (ARFID) is present, by definition anorexia nervosa and bulimia nervosa are not present. Unspecified Feeding or Eating Disorder (UFED) has no specific criteria

^a^In the ICD-11 people with anorexia nervosa who are not underweight (i.e., atypical anorexia nervosa in the DSM-5) can be classified under anorexia nervosa, and this is adopted when referring to treatment of anorexia nervosa in these guidelines (see also “Background to eating disorders and how they occur” section)

^b^Weekly compensation can include, but is not limited to severe dietary restriction, driven exercise and/or purging

^c^Descriptors include: eating much more rapidly than normal; eating until feeling uncomfortably full; eating large amounts of food when not feeling physically hungry; eating alone because of feeling embarrassed by how much one is eating; or feeling disgusted with oneself, depressed, or very guilty afterward

^d^If the criteria are no longer met, the specifier indicates whether the eating disorder is in partial or full remission

Eating disorders are common and increasing in prevalence. There is a lifetime estimated prevalence of 8.4% for women and 2.2% for men [30]. In Australia, the 3-month point prevalence is around 0.5% for low weight anorexia nervosa, 1% for bulimia nervosa and 1.5% for BED (broadly defined with ICD-criteria) and 3.2% for OSFED [including anorexia nervosa (without low weight) prevalence of 2.5%]. Furthermore, around 10% of people have recurrent binge eating [31] with rates of binge eating increasing most in people with higher weight [2]. A recent meta-analysis suggested lower rates of eating disorders but this may be accounted for by 25% of included studies being from China with large samples and generally low identification of eating disorders in these studies other than anorexia nervosa [32].

Eating disorders are also prevalent in diverse populations including men [33], across sexual and gender minority identities [34], all levels of socioeconomic status [35] and, migrant status [36]. Whilst more prevalent among adolescents and young people, they can affect people at any age including middle-aged and older adults [35, 37]. There is limited research on the experience of eating disorders in Aboriginal and Torres Strait Islander peoples. However, emerging research suggests that eating disorders are more common in Aboriginal and Torres Strait Islander adults and youth compared with non-Indigenous people [38].

Eating disorders have complex biological, social, and psychological determinants [39]. These include strong heritability and a range of risk factors that are common to and overlap with a predisposition to a higher body size, such as a personal history of trauma (see Box 3) in the formative years of life [40–42]. For people with higher weight, recommendations for weight loss by health professionals without sufficient monitoring, may be associated with the onset of an eating disorder, especially in adolescents [43].

**Box 3:** Trauma-informed care

A relationship between trauma and eating disorders is well established. Adverse experiences (e.g., emotional/physical/sexual abuse, crime victimisation, bullying) across the lifespan, but particularly in childhood are risk factors for the development of eating disorders [89–92]. Moreover, people who are at a higher weight are at greater risk of adverse experiences such as bullying and weight-related victimisation from peers, friends, parents and teachers than their peers without higher weight [93, 94].
Eating disorder treatment, in and of itself, may be traumatising for the person experiencing an eating disorder, especially when there is a lack of collaborative care and the misuse of power relations [95]. Components of eating disorder management such as weighing in a professional’s office may provoke intense anxiety, distress, and erode feelings of safety and trust. Thus, a crucial consideration for health professionals working with people with eating disorders who are of higher weight is to practice trauma-informed care through understanding the effects of actions that may be perceived as abusive, traumatic and/or triggering of previous trauma and moderating these actions as appropriate [88]. This is vital across all aspects of management of people with eating disorders who are of higher weight. For a detailed discussion of treatment principles for trauma informed care for eating disorders see Brewerton [88, 96, 97] and Trim et al. [98].
In addition to trauma-informed care, due to the high prevalence of co-occurring trauma and eating disorders, mental health professionals working with people with eating disorders who are of higher weight should also assess the need to incorporate specific trauma specific interventions (such as trauma-focused cognitive behaviour therapy or prolonged exposure) with eating disorder treatment.

Eating disorders have severe psychological, medical, community, public health, and fiscal consequences [44] with the highest mortality rates of any mental disorder [45] and high global burden—an estimated 6.6 million disability-adjusted life years [46]. Psychological comorbidity occurs in over 80% of people with eating disorders, and more specifically, in over 90% of people with bulimia nervosa or BED. Over 50% of people with bulimia nervosa or BED may have a major depressive disorder, followed by persistent depression, and around 40–50% have experienced anxiety disorders (most commonly generalised anxiety disorder). Also occurring frequently are posttraumatic stress disorder, substance use disorder (particularly alcohol use disorder), followed by a personality disorder [47]. Physical co-occurring conditions are also common. In the Udo and Grilo (2019) study [47], disorders associated with the metabolic syndrome such as hyperlipidaemia and diabetes mellitus were particularly common, as well as musculoskeletal disorders such as arthritis, fibromyalgia, and sleep problems in people with binge-eating disorder. Osteoporosis was most prevalent in people with low weight anorexia nervosa but also occurred in 6.1% of people with BED, where bowel problems (e.g., inflammatory bowel disease and irritable bowel syndrome) were also higher (around 11%) than in people without an eating disorder.

### Current status of treatment and outcomes for all eating disorders^5^

#### Psychological: first line

The first line outpatient treatment for any person with an eating disorder is an evidence-based psychological therapy delivered by an eating disorder informed and trained therapist [48, 49]. The therapies are described in Table 2. Whilst there are distinct features of these therapies, it should be noted that there are many common elements including but not limited to addressing body image (see Box 4).

**Table 2:** Overview of main psychological therapies for the management of low weight anorexia nervosa, bulimia nervosa and binge-eating disorder

	CBT-E/CBT-AN^a^ [143, 311]	MANTRA [312]	SSCM [313]	FPT [99]	IPT [100]	FBT/FT-AN [50, 51]	DBT [101]
Eating disorder indicated evidence base for use	Adults, Older adolescents Transdiagnostic/Adults with anorexia nervosa	Adults with anorexia nervosa	Adults with anorexia nervosa	Adults with anorexia nervosa	Adults with bulimia nervosa and BED	Children and adolescents with anorexia nervosa and bulimia nervosa	Adults with bulimia nervosa and BED
Theoretical model	CBT formulation & in CBT-E transdiagnostic maintaining factors	Cognitive/Interpersonal	Atheoretical	Psychodynamic formulation	Interpersonal function’s bidirectional relationship with bulimia nervosa/BED symptoms mediated by self-esteem & negative affect	Atheoretical ‘agnostic’	Understanding the dialectic of opposing views of eating disorder behaviours and their use in distress reduction
Targets	Dysfunctional eating, weight/shape (body dissatisfaction) beliefs, disordered eating	Intra- and interpersonal maintaining factors, e.g., inflexibility	Undernutrition, other ‘targets’ as personalised goals	Intra- and interpersonal maintaining factors, e.g., low self-esteem	Interpersonal (IP) problem areas: Grief, Role transitions, Role disputes, IP sensitivities	Food restriction and family eating; Other family/adolescent issues	Learning skills in: mindfulness; distress tolerance; emotion regulation; & interpersonal effectiveness
Therapy tools	Behavioural monitoring, behavioural experiments, cognitive restructuring, chain analyses	Motivational interviewing, social integration, cognitive remediation	Psychoeducation, goal-directed and supportive therapy	Exploration of beliefs/schema, interpersonal therapy, goal setting, new behaviours	Exploration of interpersonal function and eating disorder, encouraging affect, clarification, communication analysis, therapeutic relationship	Psychoeducation, externalisation of the eating disorder, family meals with initial parental empowerment to progressing to age-appropriate independent eating	Training in emotion regulation skills; ‘meaning making’ as acceptance and change; validating the worth of the individual
Mood symptoms	Core mood intolerance module in CBT-E	Emotion skills training	Symptom management	Exploration/analysis of affective-emotional experiences	Encouraging affect: acceptance; effective communication of affect; experience suppressed affects	Symptom management	Addressed through emotion regulation skills and other training

The Table is adapted from Table 2 in Hay, P. (2020). Current approach to eating disorders: A clinical update. *Internal Medicine Journal*, *50*(1), Reproduced with permission of the author (Open Access copyright)

BED = binge-eating disorder; CBT-E = cognitive behaviour therapy-enhanced; CBT-AN = cognitive behaviour therapy-for anorexia nervosa; MANTRA = Maudsley model of anorexia nervosa treatment for adults; SSCM = specialist supportive clinical management; FPT = focal psychodynamic therapy; IPT = interpersonal psychotherapy; FBT = family based treatment; FT-AN = Family Therapy for Anorexia Nervosa; DBT = Dialectical Behaviour Therapy

It was beyond the scope to include all the psychological therapies with emerging evidence for the treatment of eating disorders e.g. Integrated cognitive affective therapy [ICAT; ] and readers should not take this as an exhaustive list

^a^CBT In Guided Self-Help (CBTgsh) forms are effective for bulimia nervosa and BED

**Box 4:** Body image

Distressing and distracting eating, weight and shape preoccupations, fear and avoidance of social eating and body exposure, behaviours such as frequent body size checking/weighing, and weight/shape (negative) overvaluation are problematic symptoms across all eating disorders. These symptoms are particularly difficult for people with higher weight where health professionals and others may assume them to be ‘understandable’ or at worst, desirable. For this reason, most psychological therapies (e.g., cognitive behaviour therapy for eating disorders) incorporate specific elements aimed to reduce body dissatisfaction, address weight stigma internalisation and improve body image. In this regard, as part of the decision-making process around weight loss or weight management, the clinician should assist the person to explore the driving factors that underlie the desire to lose weight and offer options for the person to upskill in addressing weight stigma and improve body acceptance.	

##### Adults

Psychological therapies in adults include: cognitive behaviour therapy-enhanced (CBT-E); cognitive behaviour therapy for anorexia nervosa (CBT-AN); Maudsley model of anorexia nervosa treatment for adults (MANTRA), specialist supportive clinical management (SSCM); focal psychodynamic therapy (FPT); interpersonal psychotherapy (IPT); family based treatment; and dialectical behaviour therapy (DBT). Only one, CBT-E is ‘transdiagnostic’ (i.e., has an evidence-base for use in adults with anorexia nervosa, bulimia nervosa, BED and OSFED types). They are all manualised. Some have been evaluated in group, internet and self-help formats. In particular, cognitive behaviour therapy (CBT) for BED and bulimia nervosa may be delivered by primary care therapists in a guided self-help form. However, abstinence and attrition rates are superior in traditional psychological therapy and guided self-help versus pure self-help modes [49].

##### Children and youth

Family involvement in the treatment of children and adolescents at all levels of care is developmentally appropriate and best practice. A special form of family therapy with a specific eating disorder focus first developed in the UK and later the US (often referred to as the Maudsley model, family based treatment or family therapy for anorexia nervosa) is first line for children and adolescents with low weight anorexia nervosa and has been adapted for use in other eating disorders such as bulimia nervosa [49–52]. Family therapy (FBT/FT-AN) aims to establish parental management of their child’s nutritional recovery before focussing on other psychological and psychosocial issues. It has been found to be effective in a number of randomised controlled trials (RCTs) and is supported by a recent systematic review [53]. If family therapy (FBT/FT-AN) is contraindicated owing to family availability or safety concerns, then a second line treatment should be considered. High levels of family involvement in inpatient and day patient settings are usually a standard part of any program [c.f. 54–56]. Recent research has also explored the use of FBT for transition age youth (17–25 years) with anorexia nervosa, but with a more collaborative stance between parents and the young person that reflects their age [57]. The evidence-base for FBT in this age group is yet to be established.

While there is less evidence for the treatment of adolescents with bulimia nervosa in comparison with low weight anorexia nervosa, the current first line treatment for adolescents with bulimia nervosa is also FBT [58]. Family interventions for BED are yet to be studied. Alongside the published manuals for anorexia nervosa and bulimia nervosa there is also an FBT manual specific to ARFID [59] and a manualised form of CBT developed for children and adolescents with ARFID (CBT-AR) that can be delivered in individual or family based formats [60]. It is undergoing evaluation. People with OSFED are usually treated with the therapy corresponding to the full syndrome (e.g., subthreshold bulimia nervosa and bulimia nervosa).

Family specific interventions for BED are yet to be studied but there are some promising applications of IPT [61] emerging in the literature that focus on preadolescents vulnerable to developing excessive weight gain and BED. In both individual and family formats, IPT has led to improvement in internalising symptoms thought to lead to a loss of control, a symptom of BED. These are promising results given the importance of early intervention in the development of an eating disorder. In addition to IPT there is emerging evidence for CBT and DBT for BED in adolescents. CBT has been shown to be effective when compared to a weight loss treatment at both end of treatment and in the longer term [62] and DBT in reducing BED symptoms, although was not more effective than behavioural weight loss [63].

While family based treatments remain first line for anorexia nervosa and bulimia nervosa there is a need for other treatments to emerge that can specifically address other eating disorders (i.e., ARFID, BED) and non-responders in a similar evidence based way. Current recommended second line treatments for children and adolescents are noted in the next section.

#### Psychological and other: second line treatments

##### Adults

For adults who have difficulty accessing a first line therapy and/or who do not respond, or only have partial improvement, a second line treatment may be considered. Second line psychotherapies in adults include ‘third-wave’ [64] psychological therapies such as mindfulness-based therapy and Acceptance and Commitment Therapy (ACT). These have less evidence of efficacy compared to first line treatments, but may be helpful options when first line treatments have not been effective.

A psychological therapy informed by weight neutral practice and Health at Every Size® (HAES) principals (J.L Gaudiani, personal communication to author PH, August 21, 2021) has been developed with one open unpublished report (see “Psychological therapy for adults” section later in this document). It is based on an understanding that body dissatisfaction emerges in the context of weight stigma, and both are important predisposing, precipitating and perpetuating factors in eating disorders; it thus comprises weightinclusive and trauma-informed care where body acceptance (amongst others) is a protective factor.

Family interventions for adults with an eating disorder are less common and none are currently recommended as first line treatment [49]. However, some family inclusive interventions have been evaluated. The most established is Maudsley collaborative care [65, 66]. This model educates carers of adults with anorexia nervosa to support their loved one with strategies that target maintaining aspects of the illness. Parts of this intervention are also part of MANTRA, a firstline therapy (see Table 2). Other such approaches include the addition of family therapy or couple therapy alongside individual therapy [67–69] as well as group-based programs for carers.

Multiple family therapy has also been shown to be feasible with adults with anorexia nervosa [57, 70, 71]. Most studies to date report the inclusion of families in the treatment of adults with anorexia nervosa, but a recent study by Runfola et al. [72] tested a model for couple therapy specifically designed for BED in a small open trial and was found to be feasible.

Second line psychotropic medications include antidepressants, antipsychotics, psychostimulants and anticonvulsants. Their main use is summarised in Table 3. All psychotropic medications have potential to impact on appetite and body weight (though our current understanding of these effects is poor). They are seldom considered as a stand-alone treatment in eating disorders particularly because risk of relapse when discontinued and are most often prescribed as adjunctive to psychological therapy [48].

**Table 3:** Psychotropic medications commonly used in anorexia nervosa, bulimia nervosa and binge-eating disorder

Psychotropic	Medication/class	Indication	Main effects	Appetite impacts	Other and adverse effects
Antidepressant	SSRI	Bulimia nervosa; BED; Depression	Reduction binge eating Improved mood	Increase or decrease	Generally well-tolerated; may have longer term adverse effects (e.g., sexual dysfunction)
Anticonvulsant	Topiramate	Bulimia nervosa; BED	Reduction binge eating	Decrease	Sedation and neurological side effects
Antipsychotic	Second generation (e.g., quetiapine, olanzapine)	Anorexia nervosa	Reduction of anxiety & eating disorder ideation/preoccupation	Increase	Sedation and other adverse effects; appetite impacts when at an adequate weight
Psychostimulant	Lisdexamfetamine^a^	BED	Reduction binge eating	Decrease	Risk of dependency^b^; unclear when to withdraw^c^

*SSRI* selective serotonin reuptake inhibitor

^a^Only Therapeutic Goods Administration (TGA) approved medication for use in eating disorders in Australia

^b^Less than for other psychostimulants

^c^Long-term impacts on appetite and weight after withdrawing are unknown

##### Children and youth

In children and adolescents, where family therapy is not available or inappropriate, the two most common second line treatments for anorexia nervosa [49] are CBT-E for adolescents [73, 74] and adolescent focused therapy [AFT; 75]. Parent and family sessions should be offered alongside the individual sessions. Other commonly utilised interventions involving families for children and adolescents include multifamily group programs [76] and parental psychoeducation [77] as adjunctive to a first line intervention.

#### Other treatments

Behavioural weight loss intervention (BWLI) is a comprehensive psychobehavioural treatment with activity and nutrition therapy developed for people with higher weight that has since been tested as an active and as a control psychological therapy for people with recurrent binge eating and other eating disorders and found to be efficacious. In the short-term, binge eating frequency improves but in the longer term, maintenance of change is less clear [78].

Exercise and its management in general eating disorder populations (largely focusing on bulimia nervosa and low weight anorexia nervosa) is mainly targeted at reducing compulsive overexercise [79]. These interventions typically include structured physical activity under supervision (often in a group setting) and individual psychotherapy, and demonstrate improvements in depressive symptoms, skeletal muscle mass and quality of life [80, 81]. Interestingly, effects on exercise compulsion have been mixed [82]. Dittmer et al. [83] found a significant reduction in compulsive exercise in their intervention for inpatients with low weight anorexia nervosa, whilst Mathisen et al. [84] and Zeeck et al. [82], found no significant reductions compared with control groups. In contrast, Ng et al. [85] and Moola et al. [86] found that compared to a control group, people with low weight anorexia nervosa undertaking prescribed exercise reduced eating disorder symptoms, including disordered beliefs about food and exercise, and enhanced quality of life.

More recently, there have been some RCTs of neuromodulation treatments for people with eating disorders such as low weight anorexia nervosa, bulimia nervosa and BED. Treatments such as repetitive transcranial magnetic stimulation (rTMS) may aid in reducing symptoms such as binge eating and improving appetite regulation and mood [87]. As of writing this guideline they remain experimental treatments for eating disorders in Australia.

##### Psychological co-occurring conditions

Notwithstanding the need for evidence-based eating disorder treatment many people may also require psychological or other treatments for common co-occurring conditions such as major depression, anxiety disorders and/or substance-use disorder. Psychological therapy for people with eating disorders may also benefit from a trauma-informed care (see Box 3) or specific therapy such as eye movement desensitisation and reprocessing (EMDR) for post-traumatic stress disorder [88].

### Physical co-occurring conditions and consequences

Physical co-occurring conditions in people experiencing an eating disorder, with or without a high body weight, are common. In a national US sample of 36,309 adults (NESARC-III),^6^ more than half of those with an eating disorder reported at least one chronic medical condition diagnosed within the previous 12 months (54.5 ± 5.1% for bulimia nervosa and 68.6 ± 63.0 for BED), as seen in Tables 3 and 4 of Udo and Grilo [47; see further 103, 104]. Prevalence of co-occurring somatic conditions is outlined in Box 5.

**Table 4:** Assessment instruments recommended for use with people with higher weight

	Format	Useful for	Considerations for use
*Self-report*			
Eating Disorders Examination Questionnaire (EDE-Q) Version 6 [143]	28-items, with 22-items assessed on a 7-point Likert scale generate four subscale scores (Restraint, Eating Concern, Weight Concern, and Shape Concern), averaged to create a global score. Higher scores equal greater severity Specific behavioural components of disordered eating are also assessed, including binge episodes, self-induced purging, laxative misuse, diuretic misuse, and excessive exercise (not included in the global score)	Evaluating the occurrence and severity of eating disorder features in adolescents (YEDE-Q) and adults of higher weight. The YEDE-Q [144] includes age appropriate language and examples A revised version of the EDE-Q can be considered in people who have had, or are candidates for, bariatric surgery [145]	The most common and well-known tool for assessing eating disorders, the EDE-Q is faster that the EDE (interview version). The EDE-Q may overestimate the frequency of binge eating relative to the EDE The EDE-Q is not a diagnostic instrument and should not be used as an alternative to the clinical interview or the EDE in making a diagnosis Notes: EDE-Q subscale scores can still be computed provided at least half the items for the particular subscale are completed which would allow an item which may be not appropriate for a person with higher weight (e.g. Item 11—*Have you felt fat?*) to be skipped EDE-Q scores may vary for age, BMI and other features [e.g. 146–148] Available online with scoring: https://www.credo-oxford.com/pdfs/EDE-Q_6.0.pdf https://nedc.com.au/research-and-resources/show/eating-disorders-examination-questionnaire-ede-q-v-6-0-pdf-smart-form https://insideoutinstitute.org.au/assessment?started=true Accessed 10/2/2021
ED-15	Consists of 10 attitudinal items and 5 behavioural items, all rated on a 7-point Likert scale	May be useful for monitoring session-by-session change in core eating disorder features commonly targeted in treatment. Has reasonable face validity however direct validation studies in people of higher weight are lacking for the ED-15	Not suggested as a replacement for EDE-Q. ED-15 can be used as a brief complementary tool for evaluating the impact of eating disorder treatment session-by-session Available online with scoring: http://cbt-t.group.shef.ac.uk/wp-content/uploads/2019/05/ED-15-Appendix-2.pdf Accessed 25/5/2021
Binge Eating Scale (BES) [149]	16-items, each item presents three or four differently weighted statements with a final score varying from 0 to 46. Higher scores equal greater severity	Useful for the assessment of binge eating severity and BED in people of higher weight	A useful tool to rapidly screen/assess for BED however should be followed up with full clinical interview May be administered as an adjunct to the EDE-Q which does not assess for all diagnostic criteria for BED
*Diagnostic interview*			
Eating Disorder Examination (EDE) Version 17D [150]	A lengthy interview assessing core cognitions and behaviours over the preceding 3-months Regarded as a ‘gold-standard’	Most widely used measure and provides severity levels of key eating disorder features as well as generating diagnoses. Normative values are published	Available online (https://www.credo-oxford.com/7.2.html; accessed 10/02/2021) but requires training in administration Approx. 45–90 min to administer
Eating Disorder Assessment for DSM-5 (EDA-5) [151]	A semi-structured interview for feeding and eating disorder diagnosis	Assessment of DSM-5 feeding and eating disorders including bulimia nervosa and BED however validation studies are limited	A newer tool designed specifically for the assessment of DSM-5. Focus on diagnostic evaluation not related psychopathology Approx. 20 min to administer

**Box 5:** Prevalence of co-occurring somatic conditions across DSM-5 bulimia nervosa and binge-eating disorder across the BMI spectrum

Atherosclerosis	
Type 2 diabetes	
Hypertension^a^	
High cholesterol^a^	
High triglycerides	
Myocardial infarction	
Other heart conditions	
Stomach ulcer	
Epilepsy or seizure	
Arthritis^a^	
Stroke	
Sleep problems^a^	
Cancer	
Anemia	
Fibromyalgia	
Bowel problems	
Osteoporosis	
Lung problems	
Liver diseases	
Nerve problems	

Majority are uncommon (< 20% prevalence estimates)

^a^Common (20–30%) prevalence estimates in BED from Udo and Grilo [47]. Whilst diabetes, cardiovascular and metabolic conditions such as hypertension are also associated with a high BMI with or without an eating disorder, data were corrected for BMI in a later study [18] where similar findings to Udo and Grilo were found; similar findings have also been reported in two studies of children of associations between metabolic syndrome and binge-eating status [110, 111]

While higher weight has been linked to various co-occurring somatic conditions, a review by Olguin et al. [105], discussed cross-sectional epidemiologic data that showed BED was associated with diabetes, hypertension, dyslipidaemias, sleep problems/disorders, and pain conditions, and that BED may be related to these conditions independent of BMI or co-occurring psychiatric disorders. Prospective data suggest that BED may be associated with type 2 diabetes and metabolic syndrome independent of weight. BED and binge eating behaviour are also associated with asthma and gastrointestinal symptoms and disorders, and among women, menstrual disruption, pregnancy complications, intracranial hypertension, and polycystic ovary syndrome (PCOS).

The consequences of bulimia nervosa are similar regardless of BMI. These consequences include the physical effects of purging, which can affect the skin, teeth, eyes/ears and nose, throat, gastrointestinal tract, electrolytes, heart, a possible increase risk of miscarriages, and a rare risk of aspiration pneumonia [106].

People with eating disorders who restrict their dietary intake and/or engage in other behaviours such as purging may experience malnutrition resulting from poor dietary quality leading to altered body composition and body cell mass, and diminished physical and mental function and impaired clinical outcomes [107, 108] Further, the severity of the eating disorder in anorexia nervosa (without low weight) is more closely related to the amount and rapidity of weight loss and weight suppression (which may be seen also in BED and bulimia nervosa) than the actual admission weight or BMI in adolescents and physical consequences may be similar to low weight anorexia nervosa [19, 109].

#### Acute medical issues and admission

People with an eating disorder at any weight may need admission to a medical or psychiatric ward to stabilise very severe eating disorder symptoms (e.g., very frequent binge eating) and/or to reverse a starvation state or acute medical complications such as low potassium levels [see RANZCP guidelines; 112]. People may also have a co morbid medical or psychological complication requiring acute care (e.g., unstable diabetes or suicidal ideation with intent).

## Methods of guideline development

### Aim and method

#### Aim

The aim of this guideline is to synthesise the current best practice approaches to the management of eating disorders for people who are of higher weight. The focus is on the treatment of the eating disorder, with consideration of higher weight. The aim is not to address weight loss or treatment of obesity. The guideline provides guidance on providing treatment for people currently with higher weight whether or not the eating disorder developed when the person was of a higher weight.

#### Formation of the guideline development group

The National Eating Disorders Collaboration (NEDC) synthesises research evidence, clinical expertise and lived experience in national standards and workforce initiatives to build and effective, equitable and accessible system of care for all Australians. This guideline received funding from the Australian Government Department of Health. The NEDC Steering Committee agreed to auspice this guideline in 2019 and members of the Steering Committee with diverse discipline specific expertise volunteered to comprise a Writing Group. Members of the Writing Group included individuals with lived experience and/or clinical expertise and/or research expertise. At the first meetings of the Writing Group, additional members were invited into the Writing Group so representatives were included to reflect disciplines and expertise not already within the group. A wider group was then formed, namely the Guideline Development Group. This comprised the members of the Writing Group as well as additional people with lived experience who had diverse demographic characteristics (e.g., gender; Aboriginal and Torres Strait Islander status) as well as varied experiences of eating disorders, such as different diagnoses and roles (i.e., whether they had a personal lived experience of an eating disorder or were a family member or support for someone with an eating disorder). Membership was approved by the NEDC Steering Committee and NEDC National Director.

Guideline Development Group Members’ curriculum vitaes are found in Additional file 1: Appendix A along with members’ declarations of interest at the end of this document.

#### Process of guideline development

The Guideline Development Group followed the process outlined in Box 6 which is modelled on the ‘Guidelines for Guidelines’ process outlined by the National Health and Medical Research Council [NHMRC; 113]. The Group also followed the RIGHT (Reporting Items for Practice Guidelines in Healthcare) Statement for Practice Guidelines [Additional file 2: 114 ]. Decisions were made by consensus in consideration of identified evidence, and expertise and experience of members.

**Box 6:** Process of guideline development

Guideline Development Group wrote and produced a first draft of this guideline (Version 1; 2020).
Version 1 was circulated to the NEDC Steering Committee for feedback.
Guideline Development Group produced Version 2 (2021).
Version 2 was circulated for further consultation to key stakeholders including expert reviewers with professional and/or lived experience and professional organisations. A list of reviewers can be found in the acknowledgements at the end of this document. A consultation workshop was also held at the Australia and New Zealand Academy for Eating Disorders 2021 Conference.
Guideline Development Group produced Version 3 (2021).
Version 3 was circulated to NEDC members and open access to the general public on the NEDC website.
Guideline Development Group produced Version 4 (2022).
Version 4 was approved by NEDC Steering Committee.
Development Group produced Version 5 (February 2022).
Version 5 was circulated for peer review.

NEDC intends to update this guideline in 2025.

#### Research evidence

The guideline was informed by recent systematic reviews and meta-analyses as well as identified primary trials. With regard to psychological interventions for eating disorders in people with a higher weight, evidence was specifically sourced from the results of a systematic review and meta-analyses [115; manuscript in preparation]. Systematic reviews and meta-analyses were identified through a systematic literature search, existing guidelines, personal libraries of authors and additional papers identified by expert reviewers. The quality of systematic reviews and meta-analyses was critically appraised using the JBI critical appraisal checklist for systematic reviews and research syntheses [116]. The appraisal was conducted independently by author AR and contributor KP and disagreements were resolved by consensus (Additional file 3).

A full list of all the meta-analyses, systematic reviews and identified primary trials not included in a referenced systematic review used to inform this guideline is provided in Additional file 1: Appendix B. Recommendations were graded according to NHMRC categories A–D (Additional file 1: Appendix C).

It should be noted that there is a paucity of research that includes the voice of people with a lived experience.

#### Lived experience contribution

In addition to the lived experience representatives within the Guideline Development Group, further lived experience expertise was sought to co-write sections of this guideline for specific considerations for LGBTIQA+ people and Aboriginal and Torres Strait Islander peoples (see “Cultural considerations” section). We acknowledge that there is great diversity of all peoples’ lived experience, in particular, within Aboriginal and Torres Strait Islander peoples, exemplified by over 250 different languages across Australia. Moreover, we acknowledge intersectionality of people’s experiences and identities, that is, that people may belong to more than one minority group and that this may compound the difficulties they experience. Thus the views represented within this document may not capture this diversity.

#### Culturally informed practice

At the time of writing this guideline, it was apparent that there are significant gaps in the understanding and development of culturally informed assessment and treatments for larger-bodied Aboriginal and Torres Strait Islander peoples with eating disorders. When working in Australia, health professionals at all levels of experience should have received training in culturally informed practice particularly when working with Aboriginal and Torres Strait Islander peoples. This is also important to consider when working with people from culturally and linguistically diverse backgrounds and other minority groups (such as LGBTQAI+ people).

## Recommendations

### Identification and assessment

People of higher weight are at increased risk of eating disorders compared to those with lower weight [117], but due to many reasons, including poor health literacy (e.g., lack of understanding that eating disorders occur across the weight spectrum) and weight stigma in the community and in health care providers, their symptoms often go undetected and untreated (see Box 7 for a lived experience perspective) [118]. Early intervention provides the best chance of recovery when an individual is experiencing an eating disorder. Notwithstanding this, it is noted that approaches to screening have a very limited evidence-base particularly in children and adolescents, and more research is needed to establish risks and benefits [119, 120]. It is therefore imperative that eating disorder symptoms are identified and that intervention is offered as soon as possible [121] to all individuals experiencing eating disorder symptoms regardless of weight status.

**Box 7:** A lived experience perspective: identification and assessment

“My daughter’s eating disorder sneaked into our lives so quietly that we didn’t notice at first. She was 15 years old and during early puberty had gained an amount of weight that placed her into an ‘overweight’ BMI body. So, when she suggested she might try some ‘healthy eating’ we didn’t think that was a warning sign of anything more insidious. Within 6 months, she had lost more than 20% of her body weight and her period had stopped. We were very concerned as she had withdrawn from her family, wasn’t her normal happy self and her behaviours and fears around food were not normal. Our first visit to the GP was the ‘sliding doors’ moment where an eating disorder should have been identified. Instead, she was weighed and we were told, ‘It’s fine, she’s still a normal BMI. Her body probably went into shock from the weight loss and if she maintains this weight then her period will return’. Of course, that was not the case and it took another eight months before she finally received a diagnosis of Atypical Anorexia and formal treatment/refeeding began. I consider our family lucky in that despite the unacceptable delay in beginning treatment, my daughter is now fully recovered. My wish would be for families who present to their medical practitioner asking for help, to have their concerns taken seriously on Day 1.
My message to clinicians: An eating disorder cannot and should not be diagnosed by an arbitrary number on a scale…be curious and ask more questions. Please learn as much as you can about eating disorders and continue to keep your knowledge up to date with new findings/studies. Question your own biases/understanding around weight vs health and learn about Health at Every Size. Learn how to identify an eating disorder and the best evidence treatment modalities available. Eating disorders exist in all age groups, all body sizes, all genders, and all cultures. The full recovery rate from a restrictive eating disorder such as anorexia remains abysmally low so early intervention and immediate action is key to helping as many people as possible recover and go on to fulfilling lives…please learn how to be a part of the solution! For an adolescent sufferer, the carers/parents are key to doing the hard work of refeeding at home so learn how to support and empower them—this is hard work for everyone, but the rewards are huge.”
*- Julia Quin, lived experience advocate and Guideline Development Group member.*

It is important to note that binge eating, loss of control, grazing or emotional eating are not the only or even predominant eating behaviours experienced among people with higher weight [2, 122]. Dietary restriction and other disordered behaviours (e.g., use of laxatives, purging, driven or compulsive exercise, dietary supplements use or abuse) are also frequently present among people with higher weight [2]. Notably, people with higher weight experience the cognitive factors associated with an eating disorder, including overvaluation of and preoccupation with weight, shape, eating and their control, and the distress associated with these cognitions. Warning signs and clinical considerations for eating disorders among people with higher weight are outlined in Box 8.

**Box 8:** Warning signs and clinical considerations for eating disorders among people of higher weight

Warning signs and clinical considerations for eating disorders among people of higher weight include:
Recent body weight fluctuations (increases or decreases) [124].
Requests for weight loss interventions [118].
Dietary changes or severe dietary restrictions for medical (e.g., coeliac disease, allergy) or non-medical reasons (e.g., sport, veganism) [125, 126].
Presence of food insecurity [127].
Using food consumption or restriction to help regulate emotions [128].
Increases in or driven/compulsive exercise, especially where there are musculo-skeletal injuries limiting active exercise [124].
Body image concerns, especially where size and shape are influencing self-esteem (overvaluation) [124].
Depression, anxiety or substance misuse [depression/anxiety especially predictive of eating disorders in adolescent girls, not as much as in boys; [129].
Loss of menstruation or fertility in women (not due to fluctuations with puberty onset or menopause) [124].
Muscle building behaviours in males or females (i.e., intense weight training, use of sports/protein supplements, anabolic steroid use) [125, 130].
Risk for or diagnosis of type 2 diabetes (e.g., impaired glucose tolerance, signs of metabolic syndrome) [131].
Insulin misuse in diabetes (type 1 or 2) [132, 133].
Participation in elite sports or aesthetic-based industries [125, 134, 135].
Presentation with nutritional (e.g., iron) deficiency/ies [124].

When people living in larger bodies seek primary or mental health care for weight loss, assessment of eating disorder symptoms should be made. All services recommending or providing weight loss advice or programs (including bariatric surgery) should screen for disordered eating, risky behaviours such as use of unregulated weight-loss pills/supplements or laxatives, and body image concerns. All positive screens should be discussed with the individual and a more extensive eating disorders assessment should be undertaken. Health professionals in any setting should monitor any attempts at weight loss or muscle building. Short screening tools such as the Eating Disorder Screen for Primary Care [ESP; 123; see Additional file 1: Appendix D] may be also useful for this purpose. The components of a mental health assessment for eating disorders is detailed in Box 9.

**Box 9:** Mental health assessment for eating disorders

Core cognitive features.
Overvaluation of weight and shape.
Eating-related cognitions (e.g., guilt, control).
Preoccupations (e.g., with food preparation, eating, body image/appearance).
Body dissatisfaction.
Body checking.
Fear of fatness, fear of weight gain, internalised weight bias.
Perfectionism.
Food intake.
Nutritional adequacy (malnutrition is possible; nutritional status greatly impacts mood and anxiety).
Fluid intake.
Typical eating patterns/usual day.
Eating behaviours.
Past and current, and motivation to change these.
Food rituals.
Avoided foods and food sensitivities.
Triggers to eat or avoid eating (i.e ., emotional eating, binge eating, perceived restriction, rewarding oneself, sensation seeking).
Food restriction and restraint.
Weight history.
Current height and weight.
Weight changes and rate of change.
Weight-loss treatment history (especially but not only surgical interventions).
Current desire for weight loss/target weight.
Binge eating, purging or compensatory behaviour.
Type of compensatory behaviour (e.g ., laxative use, excessive exercise, diet pills, steroid use).
Frequency.
Amount.
Types of food.
Triggers to binge.
History of Medical co-occurring conditions.
Especially (but not only) metabolic syndrome, type 1 diabetes, type 2 diabetes, cardiovascular disease, sleep apnoea, musculoskeletal, polycystic ovary syndrome.
Psychosexual and interpersonal functioning.
Including important domains of functioning such as work/studies/home duties.
Eating disorder treatment history.
Psychological co-occurring conditions.
Anxiety, depression, post-traumatic stress, substance misuse all commonly co-occur with eating disorder presentations.
Personality disorders or obsessive–compulsive disorder may also be present.
Family of origin and support system.
Formative years and trauma history, especially (but not only) experiences of weight stigma and weight discrimination (i.e ., teasing, bullying and harassment or denial of access or social exclusion on the basis of weight).
Mental state assessment.
Mental health risk factor history (including self-harm and suicidality).
Psychometric assessment.

It is important to note that there is currently a lack of data regarding identification and assessment for underrepresented groups including males, adolescents, LGBTIQA+ people and people from cultural minority groups [119, 120]

Although body weight fluctuations can be a sign of an eating disorder, clinicians should not wait for body weight changes to occur before considering an eating disorder assessment.

#### Assessment of eating pathology in people with higher weight

Assessment of a person suspected to have an eating disorder should proceed in accordance with the Australia and New Zealand Academy for Eating Disorders Practice Standards 2020 [136]. Described here are particular considerations for the assessment of eating disorders among people with higher weight.

Because of wide-spread weight stigma in the community, people living in larger bodies often experience stigmatisation and discrimination because of their weight (i.e., weight teasing or bullying, negative interactions with family, friends, partners, co-workers, education or healthcare providers). Body dissatisfaction may be a natural consequence of ongoing negative evaluation rather than an irrational fear or distortion. People with higher weight also have often experienced weight-related trauma, such as bullying in high school or weight-related emotional abuse. Experiences of stigma and discrimination may lead to individuals being reluctant to talk about their weight or eating, for fear of being further shamed and/or disbelieved, and these issues must be approached respectfully, with consideration of prior negative experiences.

Disordered eating behaviours may function as a coping mechanism in the face of the trauma of persistent weight stigma. Severe dietary restraint and unhealthy compensatory behaviours may have been positively rewarded and reinforced by an individual’s social network or health professionals The person may therefore be reluctant to disclose compensatory behaviours, over-eating, or to make changes to any weight loss strategies, even though these strategies may be harmful. As opposed to attitudes of concern expressed towards smaller-bodied people engaging in dietary restriction, larger-bodied people engaging in the same or more severe degrees of restriction are often commended and encouraged to continue, with many eating disorder symptoms being perceived as helpful to the achievement of a weight loss goal. Further, where qualifying for surgery or other interventions requires the absence of eating disorder symptoms, clinicians must be cautious in their assessment of a person’s presentation.

For all people with an eating disorder, information on eating, purging and compensatory behaviours may need to be gathered from multiple sources, including family and supports, especially among children and adolescents. Eating psychopathology can impair perceptions of frequency of disordered behaviours or amount of food intake, so verification with other sources can be useful for establishing clinical status. However, for people with higher weight, it is important not to assume that the person is being untruthful. Instead, be respectful and sensitive when gathering information, even with the knowledge that a person may minimise their symptoms for fear of losing important coping mechanisms or access to interventions. The way clinicians approach questioning about eating habits and compensatory behaviours is critical to establishing a non-stigmatising and supportive therapeutic alliance. This includes respectfully seeking permission to obtain further information from family or others.

It is important not to make assumptions about a person’s eating or compensatory behaviours on the basis of weight. For example, do not assume that the person is engaging in binge eating, is untruthful about their dietary intake, or is not restricting. There are a wide range of eating disorder presentations among those living in larger bodies, including severe dietary restriction, and all possible diagnoses should be assessed before being ruled out.

A comprehensive assessment of the individual and their circumstances should be undertaken to confirm an eating disorder diagnosis and any co-occurring psychiatric or medical diagnoses, to evaluate medical and psychiatric risks, and to develop a biopsychosocial formulation. Collecting assessment information is an ongoing task as clinical issues and priorities unfold throughout treatment.

In some people with eating disorders, weight loss treatment may be contraindicated or may exacerbate their eating disorder. Where possible, attempts at weight loss or plans for bariatric surgery should be conducted in a setting to allow their eating disorder to be managed. Communication of diagnosis, medical and psychiatric risk, to other relevant treating professionals is therefore essential, especially where there are prescriptions for weight-loss treatments and/or plans for bariatric surgery. Referrals to support organisations for loved ones, family and parents are also recommended.

##### Assessment instruments

The ANZAED practice standards [137] recommend use of a psychometric assessment tool suitable for the assessment of eating disorders (using the Eating Disorders Examination Questionnaire; EDE-Q) and session by session review of progress (using the shorter ED-15). However, there is a paucity of high-quality instruments that have been validated for the full range of eating disorders among people with higher weight. Most eating disorder assessment tools have been developed and validated with predominantly low- or average-weight populations, and the language they use and concepts they measure, therefore present potential for stigmatisation and minimisation of pathology with higher weight (e.g., EDE-Q Item 11 of the shape concern subscale asks *Have you felt fat?*; and this is only considered an indicator of psychopathology in individuals of low weight). Health professionals are therefore advised to be aware of the limitations of these instruments and available to answer clarification questions in the context of a therapeutic interview. Also, the subscale scores can still be computed on the EDE-Q provided at least half the items for the particular subscale are completed which would allow item 11 to be skipped. Moreover, most validation studies for assessment measures have been conducted in predominately White female populations and therefore may not account for variations in eating practices seen in culturally and/or gender diverse samples.

Provided in Table 4 is a review of instruments recommended for use with people of higher weight. Please note that these instruments are not necessarily the most widely used nor the most frequently recommended for assessment of eating pathology in people with lower weight. Table 4 presents tools that have the most robust evidence for sensitivity, specificity and low risk of stigmatisation in the assessment of eating disorders for people with higher weight. More detailed information is provided in Additional file 1: Appendix D: Table of screening instruments.^7^

##### Assessment of anorexia nervosa and dietary restriction^8^

The use of the broader ICD-11 diagnosis of anorexia nervosa without weight criterion (as is used in this guideline) is encouraged.

For detailed information regarding anorexia nervosa see Box 10. For the assessment of anorexia nervosa among people with higher weight, it is recommended that the EDE-Q (see "Assessment Instruments" for considerations regarding inappropriate items) , is used to examine restriction, with additional questions about total and recent weight loss [19].

**Box 10:** Anorexia nervosa (without low weight)

Anorexia nervosa (without low weight; also referred to as ‘atypical anorexia nervosa’; see “Background to eating disorders and how they occur” section for definition) is a diagnosis under the OSFED category in DSM-5. This requires all criteria for anorexia nervosa are met ‘except that despite significant weight loss, the individual’s weight is within or above the normal range.’ ‘Significant weight loss’ in this context has been defined in adult men and women as 5% or more of body weight accompanied by cognitive concerns about eating and weight [175]. Significant weight loss for adolescents, however, may be less according to developmental stage [19].
Anorexia nervosa (without low weight) is commonly, but falsely, perceived as being less severe than low weight anorexia nervosa. People with anorexia nervosa (without low weight) may be just as physically compromised and experience similar or higher levels of psychopathology compared with their peers with low weight anorexia nervosa [19, 20, 176]. For people with anorexia nervosa (high or low weight) requiring hospital admission, recent and total weight loss have been shown to be stronger predictors of many physical complications, such as bradycardia, than admission weight [19].
Behaviours and outcomes that are viewed as concerning in people of low weight, such as severe restriction and weight loss, are often praised and encouraged for people with higher weight and viewed as ‘helpful’ to the achievement of a weight loss goal. All people who have lost a significant amount of weight, either recently or in total, should be assessed for an eating disorder regardless of their weight. Clinicians should be cognisant that weight gain, regardless of BMI range, may be necessary as a part of recovery (see “Nutritional and medical management” section Malnutrition). Furthermore, the use of the broader ICD-11 diagnosis of anorexia nervosa without weight criterion (as is used in this guideline) is encouraged.

Dietary restriction may be used by a person to assist in emotion regulation, or in response to experiences of weight stigma and discrimination, without weight loss, especially where restriction leads to loss of control or binge eating. For the assessment of restriction without weight loss, additional scales such as the Dutch Eating Behaviour Questionnaire [DEBQ; examines emotional, external, restraint eating; 152], or the Modified Weight Bias Internalisation Scale (WBIS-M), may also be useful.

##### Assessment of binge or loss of control eating

Although the EDE-Q is a suitable assessment tool for eating disorders among people with higher weight (see "Assessment Instruments" for considerations regarding inappropriate items), it is known that the EDE-Q measurement of binge eating and compensatory behaviours is less reliable in this population. This is because the items that pick up on frequency of loss of c ontrol eating do not contribute towards the global EDE-Q score. If binge eating or loss of control is indicated in EDE-Q items-13–15, then it is optimal to also administer the Binge Eating Scale, as this latter measure provides a better examination of behavioural indicators and distress associated with binge eating. A loss of control overeating instrument may also be used, e.g., the Loss of Control over Eating Scales LOCES [LOCES; 153. Where ‘binge’ eating appears present without loss of control, the emotional and external eating subscales of the DEBQ [152] may also be useful, especially because it has been validated in a wide range of languages.

Another form of eating associated with loss of control is grazing [154] for which brief instruments have been developed [155–157] although to our knowledge these have not been validated in people with a high BMI.

##### Assessment of exercise

Assessment of exercise among people with eating disorders is either by self-report instrument or clinical interview. A recent systematic review identified two validated instruments specifically developed for people with eating disorders, namely the Compulsive Exercise Test and the Exercise and Eating Disorders [158]. Exercise may also be assessed objectively with an accelerometer or similar, but this is not recommended clinical practice and may be triggering for people with higher weight as these are frequently used in weight loss programs.

##### Assessment of muscle dysmorphia

Muscle dysmorphia is currently characterised in the DSM-5 as a specifier of body dysmorphic disorder and with obsessive–compulsive and related disorders. Although some research has suggested muscle dysmorphia is a subtype of body dysmorphia [159], other research suggests strong similarities with anorexia nervosa, where pathological concern with muscle gain replaces pathological concern with weight loss [160]. Recent research suggests muscle dysmorphia may have validity for a stand-alone diagnosis [161].

Individuals engaging in muscle building can have very high BMI due to high muscle density but low adiposity. They are at high-risk of a wide range of disordered eating behaviours [162], and use of anabolic steroids [163, 164]. For the assessment of muscle dysmorphia*,* the Muscle Appearance Satisfaction Scale (MASS), the Muscle Dysmorphia Questionnaire (MQDMDQ), the Muscle Dysmorphic Inventory (MDI) and the Muscle Dysmorphic Disorder Inventory (MDDI) are recommended.

##### Avoidant restrictive food intake disorder (ARFID)

ARFID is a newly described eating disorder and occurs across the weight spectrum. People living in larger bodies may experience ARFID and should be assessed and managed in the same way as for people not living in a larger body. The nine-item Avoidant/Restrictive Food Intake Disorder Screen (NIAS) is an assessment instrument which have been developed for adults [165]. The Child Food Neophobia Scale (CFNS) is a good psychometric measure of food avoidance in children [166].

##### Children and adolescents with higher weight

A recent study of adolescents in New South Wales [117] found that eating disorders were more likely to be experienced by adolescents who had a BMI percentile higher than those in the lower/average weight range. Further, adolescents who met criteria for bulimia nervosa, BED, anorexia nervosa (without low weight), subthreshold bulimia nervosa, or UFED had significantly greater odds of reporting high BMI, as compared to adolescents without these disorders. Younger adolescents (Grades 7–8; 13–14 years) were as likely to experience eating disorders as older adolescents (Grades 11–12; 17–18 years), though the distribution of diagnoses among these groups was different (with older adolescents significantly more likely to meet criteria for bulimia nervosa or BED). No effects of migrant or socioeconomic status were found on the likelihood of meeting criteria for any current eating disorders when controlling for age, gender and BMI percentile.

The Youth EDE-Q [YEDE-Q; 144] has been validated among adolescents with higher weight and includes age-appropriate language. The YEDE-Q is therefore recommended for evaluating eating disorder features in adolescents with higher weight.

##### Diabetes and eating disorders

Whilst the link between type 1 diabetes and low weight anorexia nervosa is well documented, there is a dearth of literature around anorexia nervosa (without low weight) and type 1 diabetes. Adolescents with type 1 diabetes who are of higher weight are at greater risk of disordered eating than peers with type 1 diabetes but not high weight [167]. Age, diabetes duration, cultural background, family structure, insulin regimen, daily insulin dose, or glycated haemoglobin A1c concentration have not been found to be associated with risk of onset disordered eating in adolescents with type 1 diabetes, but gender and BMI have. However, high glycated haemoglobin A1c may be a marker for insulin misuse and other harmful behaviours.

Among individuals with type 2 diabetes, the prevalence of eating disorders has been estimated to be between 6.5 and 9.0% [168]. There are more therapeutic options in the management of type 2 diabetes, with many people utilising non-insulin therapies, some of which are weight-neutral (metformin and dipeptidyl-peptidase 4 inhibitors) or promote weight loss (glucagon-like peptide 1 agonists or sodium-glucose transport protein 2 inhibitors) compared to agents that promote weight gain (insulin, Sulphonylureas and Pioglitazone). The selection of medication should be made on the basis of optimising blood sugar regulation in the long-term.

Two specific instruments have been developed for screening for eating disorders among individuals with diabetes: the Disordered Eating in Diabetes—Revised [DEPS-R; 169] and modified SCOFF [mSCOFF; 170]. However, because of issues with the validity and reliability of the SCOFF for people of higher weight, the DEPS-R is the recommended instrument, particularly in type 1 diabetes [see also 171]. This is because the DEPS-R has different psychometric properties according to whether the person under examination has type 1 diabetes requiring insulin, versus type 2 diabetes. Alternatively, use of the single question ‘I take less insulin than I should’ has been identified as potentially important for detecting eating disorder symptomology in people with diabetes who are using insulin [132].

##### Weight stigma

It is acknowledged that health professionals, because they are humans who are part of society and because of their socialisation as health professionals are likely to hold both implicit and explicit bias towards people with higher weight. The Academy of Eating Disorders recommends that all health professionals evaluate their own weight stigma with an online tool [172]. While some people with eating disorders may experience improved health with weight loss, to appropriately assess and treat people with eating disorders who are of higher weight, it is recommended that health professionals adopt a weight-inclusive or weight-neutral stance, advocating for increases in health behaviours and decreases in disordered eating, instead of a focus on weight loss, which can be perceived as inherently weight stigmatising [for a detailed analysis of how weight stigma can generate stress, disordered eating and further weight gain, see 9, 173]. To examine levels of internalised weight bias in people of higher weight, the Modified Weight Bias Internalisation scale [WBIS-M; 174] may be used to document links with eating disorder psychopathology.

### Management overview

The major treatment approaches for all eating disorders have been outlined in “Current status of treatment and outcomes for all eating disorders” section. The following sections address treatment recommendations (see Tables 5, 6, 7, 8, 9, 10, 11) specifically for people with an eating disorder who are of higher weight. Treatment encompasses, but is not limited to psychological, pharmacological, nutritional and activity interventions. For all, it is important that management addresses all aspects of an eating disorder and thus will be, for the majority of people, multidisciplinary and requiring practitioners to work together as a formal or ‘virtual’ team through interprofessional collaborative practice (ICP) with each clinician practicing within the scope of their profession. ICP occurs when healthcare workers from different professional backgrounds work alongside the person experiencing the health condition, their supports, and communities to deliver collaborative care underpinned by teamwork, effective communication, and shared values [177]. This is recognised consistently throughout international and national guidelines and practice standards [48, 49, 178].

**Table 5:** Recommendation for the management of eating disorders for people with higher weight: management overview

Recommendation	Level of evidence
All treatment should be provided in the context of interprofessional collaborative practice	C

NHMRC grades range: A. Body of evidence can be trusted to guide practice e.g., meta-analyses of randomised controlled trials (RCTs) low risk of bias; B. Body of evidence can be trusted to guide practice in most situations (RCTs or other controlled studies, low risk of bias); C. Body of evidence provides some support for recommendation(s) but care should be taken in its application (moderate risk of bias in trails); and D. Body of evidence is weak and recommendation must be applied with caution (high risk of bias in trails). Full criteria in Additional file 1: Appendix C

**Table 6:** Recommendations for the management of eating disorders for people with higher weight: psychological therapy for adults

Recommendation	Level of evidence
Psychological treatment should be offered as first-line treatment approach for bulimia nervosa or binge-eating disorder (BED)	A
Cognitive behaviour therapy (CBT) for an eating disorder either in standard form or therapist guided self-help should be considered as first-line treatment in adults with bulimia nervosa or BED	B
Other psychological treatments with evidence such as interpersonal psychotherapy (IPT) and dialectical behaviour therapy (DBT) should be considered as second-line treatment options in adults with bulimia nervosa or BED	B
Other feeding or eating disorder (OSFED), unspecified feeding or eating disorder (UFED) or subsyndromal eating disorders should be treated with treatment recommended for the most similar disorder	C
Consider using therapies utilising non-dieting principles and interventions to reduce disordered eating	D
Therapies with demonstrated efficacy for the treatment of anorexia nervosa* in general, that is cognitive behaviour therapy-enhanced (CBT-E), specialist supportive clinical management (SSCM), Maudsley model of outpatient treatment (MANTRA) and focal psychodynamic therapy (FPT) should be considered as treatment options	D

NHMRC grades range: A. Body of evidence can be trusted to guide practice e.g., meta-analyses of randomised controlled trials (RCTs) low risk of bias; B. Body of evidence can be trusted to guide practice in most situations (RCTs or other controlled studies, low risk of bias); C. Body of evidence provides some support for recommendation(s) but care should be taken in its application (moderate risk of bias in trails); and D. Body of evidence is weak and recommendation must be applied with caution (high risk of bias in trails). Full criteria in Additional file 1: Appendix C

* In this guideline, the ICD 11 terminology for anorexia nervosa is adopted rather than the DSM-5 criteria. That is, anorexia nervosa (code 6B80) is used as a broad term to include all people at all body weights and without specifying the underweight criterion (sub coded in ICD-11 as 6B80.0, anorexia nervosa with significantly low body weight). See “Background to eating disorders and how they occur” section for more detail.

**Table 7:** Recommendations for the management of eating disorders for people with higher weight: psychological therapy for children and adolescents

Recommendation	Level of evidence
Psychological treatment for an eating disorder should be offered as first-line treatment approach	A
Family-based treatment (FBT) should be considered as first-line treatment for children and adolescents with bulimia nervosa and anorexia nervosa*	B
Other psychological treatments with evidence such as adolescent focused therapy (AFT) and CBT for an eating disorder should be considered as second-line treatment options in children and adolescents with anorexia nervosa* (AFT, CBT) or with bulimia nervosa (CBT)	B
Other psychological treatments with evidence such as cognitive behaviour therapy (CBT) for an eating disorder should be considered as second-line treatment options in children and adolescents with bulimia nervosa	B
Children and adolescents with higher weight should be offered a first line evidence-based treatment approach for eating disorders as those who do not have higher weight	C
Other feeding or eating disorder (OSFED), unspecified feeding or eating disorder (UFED) or subsyndromal eating disorders should be treated with treatment recommended for the most similar disorder	C

NHMRC grades range: A. Body of evidence can be trusted to guide practice e.g., meta-analyses of randomised controlled trials (RCTs) low risk of bias; B. Body of evidence can be trusted to guide practice in most situations (RCTs or other controlled studies, low risk of bias); C. Body of evidence provides some support for recommendation(s) but care should be taken in its application (moderate risk of bias in trails); and D. Body of evidence is weak and recommendation must be applied with caution (high risk of bias in trails). Full criteria in Additional file 1: Appendix C

**Table 8:** Recommendations for the management of eating disorders for people with higher weight: pharmacotherapy

Recommendation	Level of evidence
Consider using psychotropic medications with evidence in the treatment of eating disorders	B
Monitor for any non-prescribed use of medication in the context of an eating disorder	D

NHMRC grades range: A. Body of evidence can be trusted to guide practice e.g., meta-analyses of randomised controlled trials (RCTs) low risk of bias; B. Body of evidence can be trusted to guide practice in most situations (RCTs or other controlled studies, low risk of bias); C. Body of evidence provides some support for recommendation(s) but care should be taken in its application (moderate risk of bias in trails); and D. Body of evidence is weak and recommendation must be applied with caution (high risk of bias in trails). Full criteria in Additional file 1: Appendix C

**Table 9:** Recommendations for the management of eating disorders for people with higher weight: physical activity

Recommendation	Level of evidence
Physical activity interventions should focus on physical activity for positive physical and mental health benefits and away from exercising for weight or shape change	C
If compulsive exercise is present, referral to an exercise physiologist experienced in working with larger-bodied people and eating disorders populations is desirable	D

NHMRC grades range: A. Body of evidence can be trusted to guide practice e.g., meta-analyses of randomised controlled trials (RCTs) low risk of bias; B. Body of evidence can be trusted to guide practice in most situations (RCTs or other controlled studies, low risk of bias); C. Body of evidence provides some support for recommendation(s) but care should be taken in its application (moderate risk of bias in trails); and D. Body of evidence is weak and recommendation must be applied with caution (high risk of bias in trails). Full criteria in Additional file 1: Appendix C

**Table 10:** Recommendations for the management of eating disorders for people with higher weight: family and other interventions for adults, adolescents and children

Recommendation	Level of evidence
Include families and other carers when indicated for anyone with an eating disorder	B
Family psychoeducation around impacts of body and eating conversations should include modelling body image acceptance, weight stigma and a focus on health in recovery	D

NHMRC grades range: A. Body of evidence can be trusted to guide practice e.g., meta-analyses of randomised controlled trials (RCTs) low risk of bias; B. Body of evidence can be trusted to guide practice in most situations (RCTs or other controlled studies, low risk of bias); C. Body of evidence provides some support for recommendation(s) but care should be taken in its application (moderate risk of bias in trails); and D. Body of evidence is weak and recommendation must be applied with caution (high risk of bias in trails). Full criteria in Additional file 1: Appendix C

**Table 11:** Recommendations for the management of eating disorders for people with higher weight: nutrition and medical management

Recommendation	Level of evidence
Nutritional/medical guidance should minimise language that can reinforce poor self-worth and contribute to worsening eating disorder behaviours	C
Irrespective of body size, addressing malnutrition and poor diet quality is essential	C

NHMRC grades range: A. Body of evidence can be trusted to guide practice e.g., meta-analyses of randomised controlled trials (RCTs) low risk of bias; B. Body of evidence can be trusted to guide practice in most situations (RCTs or other controlled studies, low risk of bias); C. Body of evidence provides some support for recommendation(s) but care should be taken in its application (moderate risk of bias in trails); and D. Body of evidence is weak and recommendation must be applied with caution (high risk of bias in trails). Full criteria in Additional file 1: Appendix C

### Psychological therapy

#### Psychological therapy for adults

##### Evidence overview

At this time, there is no evidence to suggest that recommended evidence-based psychological treatments for eating disorders in adults of various weights (described in “Current status of treatment and outcomes for all eating disorders” section and in Table 2) are not appropriate for people of higher weight, however it is possible that they may benefit from adaptations or additions.

These psychological treatments include:

- Cognitive behaviour therapy-enhanced (CBT-E), interpersonal psychotherapy (IPT) and dialectical behaviour therapy (DBT) for adults with bulimia nervosa or BED
- Cognitive behaviour therapy (CBT), Maudsley model of anorexia nervosa treatment for adults (MANTRA), specialist supportive clinical management (SSCM) and focal psychodynamic therapy (FPT) for anorexia nervosa (without low weight)

Other approaches (e.g., BWLI) have been used for people with disorders characterised by recurrent binge eating, however these approaches are discussed only as they relate to their evidence for adults with an eating disorder and not as primary treatments for the eating disorder.

For this guideline specific research was sought for RCTs examining psychological treatments for eating disorders in adults with higher weight. A systematic review (Brennan et al. in preparation) has informed the majority of the literature presented in this guideline. A number of psychological treatments for eating disorders have been evaluated in RCTs specifically for the treatment of binge-eating disorder in adults with higher weight. These include CBT, IPT and DBT. Most of these interventions have been tested in group formats.

However, a major gap in research evidence is that RCTs in this population are nearly all confined to studies including participants with a diagnosis of BED. In particular, there were no RCTs examining the treatment of anorexia nervosa (without low weight) in people with higher weight. A further limitation was that the primary aims of most RCTs included in this review were to examine the effect of interventions on binge eating behaviours and weight. That is, higher body weight was positioned as (alongside binge eating) the therapeutic target, rather than body distress, pathological eating behaviours or eating disorder recovery. Thus, there is a need for measurements of a broader range of eating disorder outcomes (e.g., eating disorder psychopathology such as dietary restriction, body image dissatisfaction and self-induced vomiting), other psychosocial outcomes (e.g., quality of life, depression), and thorough assessment of potential harms. Follow-up in the longer term was also lacking. Further, the majority of trials of psychological interventions for people with BED (with the exception of CBT-E compared to another psychological intervention) are regarded as of low to very low quality due to high risk of bias in published reviews [e.g., 49].

##### Cognitive behaviour therapy (CBT)

CBT is the most frequently examined psychological intervention for eating disorders in adults with higher weight. Compared to wait list control groups, CBT has been shown to result in improvements in eating disorders symptoms [179–181]. CBT has also been shown to improve some body image aspects of eating disorder psychopathology (e.g., drive for thinness, body image dissatisfaction, eating concern, shape concern) relative to wait list control [182]. One study has investigated the impacts of involving spouses in CBT intervention. This did not impact on binge eating and it was associated with increased restraint [181]. CBT has been most commonly compared to BWLI and these studies are discussed below.

##### Brief and guided self-help CBT

Guided self-help (gsh)^9^ interventions have also been trialled. CBTgsh resulted in greater improvement in binge eating than BWLgsh [183]. However, CBTgsh did not improve either binge eating relative to usual care [i.e. participants’ standard individual care from primary care physician; 184] or placebo [185]. One study comparing brief CBT comprised of 6-sessions delivered over 3 or 6 weeks demonstrated similar reductions in binge eating severity and frequency in both conditions [186]. Further, CBTgsh has been evaluated and found to be effective in reducing binge eating and other symptoms in many RCTs for people with binge-eating disorder where the majority of participants are at a higher weight [see 49, pp. 620–22].

##### Other psychological interventions

Other psychological interventions that have demonstrated improvements in eating disorder symptoms relative to wait list control include behavioural activation [187], and DBT [188, 189]. In one RCT, DBT also resulted in reduced binge eating behaviours and cognitions control after a 10-week intervention compared to a wait list [189].

To our knowledge, there is one study of a weight-inclusive therapy. Gaudiani [190] has reported an open case series of 12 individuals (92% women, mean age 36.7 years, SD = 6.8) with data extracted from electronic medical records. All were perceived as living in a larger body with high levels of eating disorder symptoms and low levels of intuitive eating. Eating disorder symptoms, intuitive eating and other psychological and physical health measures all significantly improved at follow-up. Notably, body weight was not measured during therapy or reported as an outcome as this is inconsistent with the treatment [190]. Systematic reviews have also found neutral or weightinclusive approaches such as HAES to be associated with improvements in eating behaviours (i.e., reduced cognitive restraint, disinhibition and binge eating) in people of a higher body weight [24, 25].

Other psychological treatments have also been compared to CBT. The one study comparing DBT to CBT reported no between group differences in eating disorder psychopathology at post-treatment, but the CBT group demonstrated greater improvements at follow-up. In addition, the CBT group demonstrated greater improvements in binge eating post-treatment, but no differences between treatments at follow-up [191]. One study comparing CBT to IPT demonstrated that both treatments resulted in comparable improvements in binge eating frequency and cessation post-treatment, and while there were minor increases in binge eating frequency at 12-month follow-up, both groups continued to demonstrate reductions in binge eating compared to pre-treatment. Both groups demonstrated reductions in pathological dietary restraint, CBT had larger effects post-treatment, but groups were equivalent at 12-month follow-up [192]. Hilbert et al. [193], have reported effects which were sustained in the longer-term, up to four years. A further study compared IPT, BWLI and CBTgsh and found that post-treatment all treatments produced improvements in binge eating frequency and cessation, and eating, shape and weight concerns, but that at 2-year follow-up IPT and CBTgsh resulted in greater binge eating remission rates, and BWLI resulted in greater cognitive restraint [78]. For people with a higher frequency of binge-eating, IPT appeared to be more effective than CBTgsh and BWLI [78].

##### Adapted treatments including CBT and BWLI for eating disorders characterised by recurrent binge eating

Psychological interventions have been most often compared to or used consecutively with BWLI. BWLI however aims to both reduce binge eating and elicit weight loss [see Box 2; 194]. While BWLI and CBT share some common characteristics (e.g., self-monitoring, use of behavioural strategies to reduce binge eating episodes) the primary goal of CBT is treatment of the eating disorder, and restraint is considered a maintaining factor and therefore a target of CBT interventions is the reduction of restraint. Furthermore, most RCTs have found CBT to be more effective than BWLI in improving eating disorder symptoms (e.g., binge eating) and in some cases achieving remission of binge eating [195–197]. However, in some studies there is no difference between treatments at 6-months [199], or 12-month follow-up [196], and in other studies, between-group differences are greater at 6-month follow-up [197, 198]. 

Only a few studies comparing CBT and BWLI have measured other eating disorder psychopathology such as body image concerns [194]. One found that CBT and BWLI resulted in similar improvements in eating, weight and shape concern [196]. Conversely, Grilo et al. [197] found that neither of these treatments produced an effect on these variables nor on restraint. One further study found BWLI to increase restraint relative to CBT [78], and another that CBT resulted in greater improvements in eating, weight and shape concern, but not restraint, relative to BWLI [202]. Only one study comparing CBT to BWLI has conducted long-term follow-up. At post treatment, CBT resulted in greater improvements in binge eating frequency and BED diagnosis [196]. However, there were no differences between groups at 6-year follow-up [199].

Other studies have evaluated sequential CBT and BWLI. For example, in one study participants who responded to CBT (i.e., improved eating disorder symptoms) were then offered BWLI while those who did not respond to CBT were offered IPT. The responders offered BWLI intervention demonstrated further improvements in binge eating and further weight loss, while the non-responders offered IPT demonstrated increased binge eating and small increases in weight [179]. In a second study, participants received either CBT, BWLI, or CBT followed by BWLI. There were no differences in binge eating remission between groups post treatment, but at 6-month follow-up the CBT alone group demonstrated significantly greater binge eating remission than BWLI alone or in combination with CBT [197].

A recent study compared BWLI to a stepped care model in which non-responders to BWLI were stepped up to CBTgsh. Both conditions demonstrated significant improvements in binge eating remission and frequency, with no difference between groups [200].

One RCT has tested an integrated BWLI with CBT-E in a transdiagnostic group with BN, BED and OSFED [201].^10^ There were significant within group reductions in eating disorder symptoms but only one between group difference for main eating disorder psychopathology outcomes. This was an increased binge eating remission rate with the integrated intervention at one year compared to CBT-E. Secondary outcomes are yet to be published [206]. Cooper, Calugi and Dalle Grave [203] have also proposed an integrated treatment but this is as yet untested.

A systematic review of mindfulness-based interventions for people of higher weight found that mindfulness-based interventions resulted in a significant decrease of binge-eating disorder symptoms, when compared with control [204]. However this was an exploratory analysis due to the limited number (i.e., three) of studies available.

##### CBT and other dietary and non-dietary interventions

CBT in combination with dietary interventions, such as low calorie diets (LCDs) or nutritional counselling, has not demonstrated advantages over CBT alone with regards to eating disorder symptoms [205, 206]. In contrast, combining CBT with inpatient treatment for obesity has been shown to improve binge eating episodes, relative to inpatient treatment alone, at 12-month follow-up [207]. BWLI has also been compared to non-dieting interventions (promoting improvements in health behaviours and body image without intentional weight loss). Both resulted in improvements in binge eating severity [208]. More recent weight neutral or weight-inclusive approaches, such as HAES, have shown improvements in eating behaviours (i.e., reduced cognitive restraint, disinhibition and binge eating) however such interventions have no published evidence to date in people with eating disorders [24, 25].

##### Clinical considerations

There are some important issues specific to the treatment of people with eating disorders who are of higher weight that clinicians should be aware of.

###### Approaches for people with anorexia nervosa/restrictive eating disorders

Resumption of menses has been identified as an important treatment goal for females with restrictive eating disorders as it is a factor contributing to improved bone mineral density [209]. Restoration to pre-morbid weight, even if this is at a relatively high BMI, may achieve the most complete and long-lasting recovery [210]. However, research on weight restoration for anorexia nervosa among people living in larger bodies is currently lacking but has been noted as a priority for future research. 

###### The value of in-session collaborative weighing

Evidence-based psychological therapies for eating disorders all stress the importance of in-session weighing. This is to monitor weight for safety reasons (e.g., in the case of anorexia nervosa and related disorders to make sure the person is restoring weight and/or not losing weight) as well as for the purpose of achieving cognitive change. However, when working with people with eating disorders who are of higher weight, the value of in-session weighing should be carefully considered, and the benefits evaluated against the risks of any possible negative consequences. For some people with higher weight, in-session weighing is recommended but options such as blind weighing can be considered. Again, this issue should be raised by the therapist and discussed openly with the individual before treatment begins. Where malnutrition is suspected (for example after prolonged dietary restriction or significant weight loss, regardless of current body weight) or there are medical co-morbidities present, a dietitian and a general practitioner should be closely involved in care and may use weight change as a marker of nutritional status. However, as above, weight change can be monitored without the person being aware of their weight if that is their preference.

###### Weight stigma

As highlighted in “Weight stigma” section ‘weight stigma’, therapists working with people experiencing eating disorders who are of higher weight need to be aware of the negative effects of weight stigma, and that fact that they, themselves, may be influenced by weight stigma which may make it more difficult to focus treatment on the person’s eating disorder rather than on their weight. Further training and supervision by a skilled clinician in this area may be helpful.

#### Psychological therapy for children and adolescents

##### Evidence overview

There is no evidence to suggest that current evidence-based treatments for eating disorders in children and adolescents are not appropriate for people with higher weight. As outlined in “Current status of treatment and outcomes for all eating disorders” section FBT is the first line treatment for anorexia nervosa and bulimia nervosa for this age group, with second line treatments for anorexia nervosa being adolescent focused therapy (AFT) and CBT-E. CBT-E is also considered a second line treatment for bulimia nervosa. However, guidelines vary as to how strongly these second line treatments are recommended [49, 52]. For BED, adult treatments are recommended [49] and for ARFID there is no recommendation, but CBT is noted as promising [52]. As noted earlier, an evidence-base for specific psychological interventions or modifications to current evidence-based treatments for those with higher weight does not exist.

##### Clinical considerations

Modification of current evidence-based treatment for young people with and eating disorder and who are of higher weight is not yet indicated and treatment directives such as weighing the person experiencing the eating disorder in session should be followed. However, clinicians should proceed with sensitivity and judgement mindful of the potential for increasing shame and the impact of weight stigma and how this may impact on the young person’s and family experience. Some aspects of public health campaigns focussing on reducing childhood obesity (e.g. weighing of children in school) may trigger the development of an eating disorder in vulnerable young people. A common clinical impression from parents is the lack of recognition they can receive for their child’s difficulties and the delay this creates in receiving help. Young people on the other hand, often feel a sense of failure to be ‘seen’ as sick enough because of their weight. These and other related experiences should be recognised and integrated into the young person and family’s treatment to improve engagement.

### Pharmacotherapy

#### Evidence overview

There are no medications developed for the treatment of people experiencing an eating disorder who are of higher weight where the primary outcome is improvement in eating disorder symptoms and/or behaviours. There are also no medications recommended in current general guidelines [48, 49] as first line in the treatment of an eating disorder. Whilst RCTs have found evidence of efficacy for some medications, for example, SSRIs particularly in people with BED or bulimia nervosa, effects are not sustained when the medication is withdrawn [48]. There are two groups of medications that are, however relevant to the scope of this guideline:

1. Medications that may be used for people with eating disorders. These are not recommended as ‘first-line’; they are most often used as adjunctive treatments.
2. Medications used to reduce appetite with potential to impact on eating disorder treatment.

It is also important to acknowledge that research in the use of medications in BED has been biased towards participants of whom either all or a very high proportion were people with higher weight. For example, in the NICE [49] guidelines all reported RCTs of pharmacological therapies in BED are of participants with a high BMI (> mean 30). Covertly or overtly, weight loss/maintenance in these trials is often presumed to be a positive treatment outcome.

Furthermore as we have noted medications are most often used as adjunctive treatments where they may enhance the efficacy of the psychological therapy however the present state of evidence is insufficient to recommend routine use in addition to psychological therapies.

#### Medications that may be used for people with eating disorders (see also Table 3)

##### Lisdexamfetamine

This is a stimulant approved in Australia for treatment of BED. It is not approved for appetite suppression but has this effect. It is cautioned and is a relative contraindication in people with histories of substance use disorder and/or who are in the underweight range, in a state of weight loss or weight suppression. This is particularly true for people with past or current anorexia nervosa and some people with bulimia nervosa. Most efficacy trials have included a majority of people with a high BMI.

##### Antidepressants

The majority of evidence for efficacy of antidepressants for people of a high BMI and an eating disorder is confined to BED and is of low to very low quality. They are inferior to CBT, and there is insufficient evidence they will enhance CBT or other psychological therapies. Relative risk for remission is 1.39 (0.92–209) in four studies to 12 months [49; Table 275]. Most evidence is for fluoxetine (up to 80 mg per day in BED; 60 mg per day in bulimia nervosa). Antidepressants may be considered for bulimia nervosa and BED where there is co-occurring depression or difficulties accessing psychological therapy. Antidepressants may reduce appetite in the short-term and/or be associated with reduced appetite in the longer term.

##### Anticonvulsants

There is limited evidence for the use of topiramate in bulimia nervosa and BED. It is poorly tolerated with several adverse effects including weight loss, sedation and neurological symptoms [211]. One RCT of lamotrigine [212] in people with BED with higher weight reported a very high placebo response, similar to the active drug effect for binge eating.

##### Antipsychotics/mood regulating agents

All antipsychotics and mood regulating agents, but particularly second-generation medications such as olanzapine, may cause increased appetite, weight gain and exacerbate conditions associated with a high BMI such as metabolic syndrome and type 2 diabetes [213]. They also have a range of other problematic adverse effects such as sedation. When prescribed for a person with higher weight, one that is least likely to impact on appetite should be considered [214]. If there is severe weight gain, a change in antipsychotic/mood regulating agent should be considered as people may develop an eating disorder or exacerbation of eating disorder symptoms as a consequence.

##### Other agents

Atomoxetine is a selective norepinephrine reuptake inhibitor. Evidence in eating disorders is limited to one trial in BED where it was associated with binge eating reduction [215]. Similarly Armodafinil, a psychostimulant has been found in one trial of BED to reduce binge eating [216]. Finally, dasotraline, a new agent with dual dopamine and noradrenaline reuptake inhibition, has been found in two RCTs to reduce binge eating in people with BED [217, 218]. It also reduced appetite in people with higher weight [219]. None of these agents are approved for use in eating disorders in Australia.

#### Medications used to reduce appetite

The weight loss medication orlistat has been trialled in people with BED who are of higher weight but it has poor tolerability and there have been reports of its abuse in people with bulimia nervosa [220]. It has not been approved for use in BED in Australia. Medications such as metformin, insulin and semaglutide may alter food consumption and consideration of this, and potential for non-prescribed use needs to be applied in the care of a person living with a higher body weight and an eating disorder.

### Physical activity

#### Evidence overview

While there has been much research on exercise interventions for people of higher weight, few studies directly examine physical activity in the treatment of eating disorders among people with higher weight. However, a range of physical and psychological benefits (e.g., improved self-perception, body image and mood) have been found in studies involving structured and tailored exercise interventions in eating disorder populations. Such exercise is commonly part of a broader lifestyle, BWLI or LCD intervention and may take place in the workplace, where people spend a large portion of their time. It includes the implementation of walking routes, team exercise classes, improvements in cafeteria/vending machine options and team psychoeducation [221]. It is likely that these programs vary greatly in their weight-centrism and potential to reinforce weight stigma. As this literature does not directly assess or refer to underlying eating disorder psychopathology caution is needed when translating such findings to eating disorder populations where exercise can become compulsive and used in an attempt to compensate for binge eating episodes. Meta-analyses have consistently found that exercising for predominately weight and shape reasons is likely to be associated with the onset and/or exacerbation of an eating disorder [222–224].

Levine et al. [225] looked at the effects of a 6-month exercise intervention in women with BMI > 30 and BED and found significant reductions in binge eating symptomatology in the treatment group compared with control, but no difference in effect on depressive symptomatology. Pendleton et al. [226] trialled exercise-augmented CBT in BED and also found significant reductions in binge eating symptomatology post-treatment. McIver et al. [227] found a yoga intervention significantly reduced self-reported binge eating in higher weight individuals as compared with a wait list control group who did not improve on any measure at post-test. 

#### Clinical considerations

The literature has been evaluated in conjunction with clinical expertise to inform this guideline, and further research is needed to build a solid evidence-base. Primary treatment goals in this population should be psychotherapeutic and focus on self-acceptance and the development of a healthy relationship with exercise [228]. Emphasis should be placed on the physical and mental health benefits of regularly engaging in exercise [229], and more importantly, improvements in self-perception and positive wellbeing [230, 231] rather than a narrow focus on weight. Whether conducted with a normative or general eating disorder population, research consistently demonstrates multicomponent approaches including psychoeducation to be more broadly effective for improving physical and psychological health than behavioural changes alone [232]. What constitutes ‘effective’ will also depend on the individual and their goals. Exercise recommendations rarely consider current fitness levels, impaired mobility, or existing mental health concerns, such as eating disorders. Wherever possible, people with an eating disorder and are of higher weight should be engaged with a multidisciplinary team and any exercise or physical activity program should be closely monitored by a trained eating disorder and exercise professional, begin at an appropriate intensity and increase slowly over time in a graded fashion [228; see Box 11].

**Box 11:** Exercise in eating disorder treatment

Exercise can be an effective intervention for many psychological health issues [e.g., depression; see 233], however, has often been overlooked as a potential adjunct intervention to eating disorder treatment. A systematic review by Cook et al. [228] outlined 11 core themes describing techniques that have been successful in using exercise as an adjunct in eating disorder treatment. These themes are: “employ a team of relevant experts; monitor medical status; screen for exercise related psychopathology; create a written contract of how therapeutic exercise will be used; include a psych-educational component; focus on positive reinforcement; create a graded exercise program; begin with mild intensity exercise; tailor the mode of exercise to the needs of the individual; include a nutritional component; and debrief after exercise sessions” [228; p.1408]	

Notably, clinical judgement should be utilised when dealing with vulnerable populations. For people with eating disorders exercise can be pathological or unhelpful in nature or frequency, thus exercise interventions for those people with higher weight need to take a different approach. People with higher weight may face additional challenges when attempting to implement exercise interventions due to current and/or previous experiences of weight stigma, prejudice and discrimination. Notably, exposure to exercise environments (such as gyms) very often involve exposure to weight stigmatising environments.

### Family and other interventions for adults, adolescents and children

#### Evidence overview

The evidence-base for family interventions specific to people with an eating disorder who are of higher weight is extremely limited and no interventions developed for children and adolescents with eating disorders note any specific treatment adjustments for young people with higher weight. Further, none of the adult family interventions reported above (“Current status of treatment and outcomes for all eating disorders” section) specifically address or recommend an augmentation for people with eating disorders who are of higher weight.

People with anorexia nervosa (without low weight) may live, or have previously lived in a larger body. While FBT (see “Context” section, Table 2) was initially developed for people with eating disorders who are in an underweight range, there is some evidence for its application to individuals with anorexia nervosa (without low weight) without augmentation of the model [234]. However, a recent qualitative study of practitioners applying FBT to people with anorexia nervosa (without low weight) identified a lack of clarity on appropriate weight targets, the use of the weight chart in treatment and difficulty activating urgency in the parents [235]. These are all critical aspects of FBT for anorexia nervosa.

There is a body of literature around the negative effects of weight/shape and eating conversations, from familial, peer and other sources, for children and adolescents. Amongst many psychological consequences is an increased risk of eating problems [236, 237].

#### Clinical considerations

Clinicians should implement evidence-based treatment interventions for people with eating disorders who are of higher weight as recommended and continue to involve families in treatment. At the least, psychoeducation of families and supports are needed. This would include emphasising that nutrition is critical, providing information about what constitutes normal eating, and the way in which malnutrition impacts the brain and makes body distortion/fear of weight gain worse. Nutritional recovery often leads to weight gain, regardless of the person’s initial starting weight. Similarly, nutritional recovery commonly results in improved cognitive function, although improvements in eating disorder thinking often lags behind other changes. It is ideal to deliver psychoeducation on psychosocial impacts of an eating disorder when in a larger body. This may include how families manage their own weight stigma and conflicting advice from health professionals regarding the desirability of weight loss.

Structured support from family/supports to facilitate regular and adequate eating will assist with eating disorder cognitions and returning a normal eating pattern. This may include the responsibility of purchasing of food, preparing of meals, and support at mealtime. Families should be encouraged to check in with their own assumptions about body shape and size so their loved one can focus on recovering from the eating disorder, rather than on a fear of returning to or maintaining larger body size. Families should be encouraged to use body neutral and body positive talk. Health professionals reflecting on their own use of body negative talk and overvaluation of shape and size is important. Changing our own language and thoughts can model body image acceptance and a focus on health in recovery.

Families should be encouraged and supported to develop distress tolerance skills for both themselves and the person with the eating disorder rather than using disordered eating behaviours to reduce distress. Encouraging families and supports to consider social media usage in the home and supporting media literacy in the eating disorder affected person is also likely to be helpful. Evidence-based resources include Mental Health First Aid, including online resources, and eating disorder specific training.

It is important to note that families are also expected to be active in the second line individual treatments (CBT-E and AFT) discussed earlier. While parents are not present for every session their role is defined and participation critical. Similarly, the inclusion of parents in any emerging treatment for children and adolescents is going to be essential given the importance of both the relationship and family context. IPT for BED as discussed in “Psychological: first line” section is an exemplar of a novel treatment also being delivered in a family-based format [238].

### Nutritional and medical management

#### Evidence overview

The research evidence for nutrition care for people with an eating disorder who are of higher weight is covered above in the sections on BWLI, CBT and other dietary interventions, and exercise. There is no evidence to support any dietary intervention as stand-alone care for treatment of an eating disorder. Nutritional assessment and management of nutritional care in larger-bodied people with eating disorders is best provided with the support of a dietitian.

#### Clinical considerations

The nutritional and medical management of person with an eating disorder who are of higher weight must address both the eating disorder and any other health needs of the individual. This may include nutritional complications of the eating disorder, and the nutritional needs of physical and mental health co-occurring condition. A priority is the nutritional management of medical conditions such as type 1 diabetes, with awareness that an eating disorder may complicate dietary management.

#### Malnutrition

Addressing malnutrition is essential for preventing life-threatening and longer-term complications in those with a restrictive or other eating disorders [19, 239]. Malnutrition is generally defined as a BMI < 18.5 kg/m^2^ or unintentional weight loss of ≥ 5% with evidence of suboptimal intake resulting in subcutaneous fat loss and/or muscle wasting regardless of BMI [240]. However, *intentional* weight loss, or being in a state of ‘weight suppression’ (i.e., a discrepancy between one’s highest adult weight and current weight), should not preclude a diagnosis of malnutrition in someone with an eating disorder, and identifying malnutrition beyond current weight, with assessment of percentage of weight loss is recommended by the American Academy of Paediatrics, American Society for Parenteral and Enteral Nutrition, Academy of Nutrition and Dietetics and the Society for Adolescent Health and Medicine [209, 241, 242]. However, identification and assessment are only the first steps in the nutritional rehabilitation process required to reverse malnutrition.

There are numerous clinical guidelines outlining the best evidence-based strategies for treating malnutrition and improving dietary quality [243], which may help guide the nutritional interventions for malnourished people with an eating disorder who are of higher weight. However, it is important to ensure that nutritional rehabilitation not only addresses immediate nutritional needs to prevent further weight loss, but also the body’s need for physical repair of any damage incurred during dietary restriction and other eating disordered behaviours resulting in malnutrition. A person’s body weight may need to increase to allow for this physical repair and restoration. This may be difficult for the person with an eating disorder to accept when their sense of identity is closely linked to their appearance, and they have been striving to lose weight. They also will encounter, and be distressed by, the negative consequences and stigma (perceived or actual) of a higher weight.

#### Micronutrient deficiencies

People with higher weight may have micronutrient deficiencies (e.g., zinc, iron, vitamin D, B-group vitamins, etc.) due to low diet quality and potentially reduced bioavailability [244–246]. Moreover, eating disorders may potentially result in micronutrient abnormalities or deficiencies as a result of dietary restriction and eating disorder behaviours (e.g., vomiting) leading to medical complications.

#### Other medical problems

While higher weight is associated with a multitude of medical and psychological conditions, this section deals with the management of medical conditions in people with both an eating disorder and with higher weight. Eating disordered behaviours in people with higher weight may also lead to a range of medical complications that require intervention. As previously mentioned, people of any weight with BED are at risk of medical complications such as type 2 diabetes, hypertension and dyslipidaemia [47]. These conditions often require specific dietary restriction and modification. Although traditional dietetic interventions for people with higher weight with such medical conditions have promoted the primary goal of specific dietary modification for weight loss [247–249], these effects appear short-term, and may bring unhelpful consequences such as weight regain, binge eating, body dissatisfaction, eating disorders and low self-esteem 250, 251. Further, health gains may be achieved with improved diet quality alone [252, 253].^11^

Nutritional guidance on management of such medical complications therefore needs to be aware of language and avoid messaging that can reinforce poor self-worth, feelings of failure and stigmatisation, which can all contribute to worsening eating disorder behaviours rather than reducing the medical complications. Individualised nutrition counselling and dietary adaptations to manage medical co-occurring conditions are important. This may include non-weight loss focussed dietary approaches and HAES approaches, which incorporate directly targeting unconditional body shape and size acceptance, and encourage physical activity and eating for well-being, including eating according to appetite, decreasing vulnerability to external stimuli and coping with emotional eating. A systematic review of randomised and non-randomised studies examining HAES interventions for management of BMI suggests that HAES, focusing on more comprehensive health outcomes rather than weight loss alone, may be effective for improving some cardiovascular outcomes, but further studies examining the effect on blood glucose and blood pressure are needed [24].

The presence of binge eating, purging and other eating disorder behaviours complicates the management of diabetes. Goebel-Fabbri [258] has written a practical guide to management of eating disorders and type 1 diabetes, some of which is also relevant for management of type 2 diabetes. A clinical guideline for disordered eating and eating disorders in adults with type 1 diabetes (aged 16 years and over) produced by Queensland Health is also available [259]. Polycystic ovary syndrome (PCOS) is also associated with an increased risk of disordered eating [260, 261] and care needs to be taken not to exacerbate body image issues and eating disorders in this group of women [262].

In the case of bone health, although people with higher weight are thought to have higher bone mineral density (BMD), they appear to have an increased risk of fractures at some sites [263]. If severe dietary restriction and malnutrition is layered on top of this, leading to inadequate calcium intake and potentially a fall in oestrogen in females, this may place the individual at an increased risk of fractures. Current research suggests adults with anorexia nervosa (without low weight) have significant bone deficits, while adolescents with anorexia nervosa (without low weight) have BMD scores higher than adolescents with anorexia nervosa who are underweight [264], with their BMD potentially protected by their premorbid higher weight.Further, in atypical anorexia nervosa, lack of current low weight or amenorrhoea does not prevent reduced vertebral strength [265], and should be considered as a potential concern in all individuals with an eating disorder who have a history of severe dietary restriction. However, findings have been inconsistent [e.g., 266].

#### Bariatric surgery

It is important to assess for an eating disorder in people with higher weight attending for bariatric surgery assessment, as the prevalence is high [267]. People with a history of eating disorders also often plan to undergo bariatric and/or cosmetic surgery [268]. Additionally, although binge eating and psychological conditions like anxiety and depression may improve in the short-term following bariatric surgery, they may restart over the longer term [269, 270]. Continuing psychological support may improve outcomes in the longer-term from bariatric surgery [271]. However, the data are quite mixed and most point to the need for an improved understanding of who will develop loss of control eating after surgery as opposed to prior to surgery.

### Other psychiatric therapy for co-occurring conditions (e.g., bipolar disorder, psychosis)

Both BED and bipolar spectrum disorders are frequent co-occurring conditions in people with higher weight, and experiencing both BED and bipolar disorder concurrently is associated with more severe eating behaviours and psychopathology [272]. Furthermore, it is suggested that approximately 10% of people with schizophrenia have BED [273]. People with such psychiatric co-occurring conditions often require antipsychotic medication which is associated with rapid weight gain and metabolic abnormalities as detailed earlier [274–276]. These medications are known to increase appetite, decrease satiety and increase cravings for sweet foods and drinks, as well as contribute to disordered eating habits, such as only eating one main meal each day [277, 278]. Mood stabilisers (e.g., lithium) and anticonvulsants (e.g., valproate) can also have weight gain effects [276]. In people with an eating disorder who are of higher weight who are also taking antipsychotic medication, it is important to be aware of the risk of onset of disordered eating and eating disorders in this context.

### Cultural considerations

Evidence-based knowledge of cultural considerations in the management of eating disorders is in its infancy. To our knowledge there are no studies that specifically addresses cultural considerations for the treatment of eating disorders for people with higher weight. The following paragraphs are derived from research pertaining to cultural considerations for the treatment of eating disorders (at any weight) as well as lived experience and clinical expertise. The below groups were chosen as salient groups that are under-represented in the eating disorders literature and treatment services within the Australian context, however such considerations may be relevant for similarly under-represented and disadvantaged groups across the international context. See a recent systematic review by Acle et al. [279] for empirically derived guidance on how to effectively address culture in eating disorder treatment among racial/ethnic minorities. A lived experience perspective is provided in Box 12.

**Box 12:** A lived experience perspective: cultural considerations

“It started when I was in high school. I was 15. I was not coping at home. I was not coping at school. My fixation with food intake or lack of it, was something that I could control. After passing out a number of times, I was tested for everything from diabetes to possible brain tumours. The doctors just kept saying that they could not find anything wrong with me.
Throughout this time, I had body maintenance and being male, no-one could acknowledge or diagnose that I had an eating disorder. In Year 10, after collapsing in the playground at lunch, I was transferred to hospital by ambulance. It was a teacher that escorted me in the ambulance that made a comment to the paramedics that she thought I may be experiencing an eating disorder. At the hospital things started to change. I was not poked and prodded at, rather the doctors and nurses started talking to me about my thoughts, feelings and experiences. I was diagnosed with bulimia. I was given the label—but no-one really knew how to work with me. ‘I never worked with a male before’, 'it is not common in boys’, and 'you don’t meet the case studies that I have seen in research’ were common statements that the medical staff and allied health professionals like psychologists and social workers stated to me. The health workers struggled at times to engage with me. I already felt odd and out of place and then to be told that I was an 'anomaly' was so hard. I remember being given a booklet about eating disorders. I could not see anything in it that related to me. It told me that my periods would stop, and my breast development would be disrupted. Everything that a 16-year-old boy needs to be aware of. Being an Aboriginal male there was also very little information about eating disorders in community or any real cultural support offered. I am not sure that much has changed over the last 30 years. What I would like to see in the future is more recognition that eating disorders affect all people and is predominate in all cultures. It is important for young men to understand that eating disorders can affect them and to have resources and support tailored to meet their unique needs. It is also pertinent that health professionals work in culturally safe ways to support Aboriginal & Torres Strait Islander people and their families throughout their diagnosis and treatment journey. So for me there were at least four assumptions that were wrongly made that prevented me from getting help earlier. Firstly, it was about who has/ or can develop an eating disorder. The second was that that health professionals were looking for a certain body size. Thirdly, there was a cultural barrier, nothing that was Aboriginal-specific as a resource and lack of cultural awareness or cultural competence. Finally, there was also a gender barrier that involved both the fact the professionals that I worked with mostly non-indigenous females and I was a young Aboriginal male who didn’t meet the stereotypes of those with an eating disorder, as well as literature that aimed for female clients. It is now time to make changes to stereotypes and becoming more culturally alert to the diverse range of clients.
*AJ Williams-Tchen, lived experience advocate and Guideline Development Group Member.*

#### Men with eating disorders

Historically perceived as disorders of women, eating disorders can affect people of any gender. While there has been an under representation of males in eating disorder research [280], it is estimated that one third of people reporting eating disorder behaviours in the community are male [281]. Males account for approximately 30% of people with bulimia nervosa, 57% of people with BED, 55–77% of people with OSFED [subtype-dependent; 282] and 67% of ARFID [283].

In comparison with women, men are more likely to have a history of higher weight prior to their eating disorder, accompanied by weight-related bullying [236, 284]. In addition to weight stigma and the stigma associated with having a mental illness, males may experience stigma associated with having a ‘female’ disorder which may present as a barrier to seeking and engaging in treatment [33, 285]. Men also experience a later age of onset [281] and higher rates of co-occurring psychiatric conditions [286]. Despite this, research shows that health professionals are less likely to recognise eating disorder behaviours in males as a mental health problem, and this less likely to offer treatment [280, 287].

In Westernised society, the majority of males report desiring a lean muscular physique [288, 289] as opposed to a ‘thinner’ physique often desired by women [290]. This pursuit of a muscularity may manifest in a wide range of eating disorders behaviours including misuse of anabolic steroids [163, 164].

While men can experience all eating disorder diagnoses, some differences in eating disorder psychopathology have been noted across genders. Men are less likely to report a loss of control over eating, despite having similar rates of objective binge eating to women and are more likely to engage in compulsive exercise for emotion regulation [291]. The management approaches described throughout this guideline are not gender-specific, however health professionals may need to hold additional considerations in mind when working with men such as the importance of exploring and challenging ‘masculine’ concepts of strength, power and control for greater treatment engagement [292]. Clinicians are also encouraged to be attuned to how men express and communicate (often gendered) emotions including distress, anger, grief, irritability, anxiety and sadness. For additional information on considerations for psychological therapy when working with men with eating disorders see Bunnell [293].

#### Aboriginal and Torres Strait Islander peoples

Owing to the limited evidence for the treatment of eating disorders for Aboriginal and Torres Strait Islander people, health professionals working with people experiencing eating disorders and their families, should apply caution when applying this guideline to Indigenous peoples and recognise there may be a need to customise or tailor current treatment and communication approaches to accommodate their culturally diverse needs, resources and expectations.

It is suggested that health professionals refrain from using clinical language and overreliance of health literature in awareness that some Indigenous peoples have lower literacy levels and/or English as a second language, lower health literacy, and lower mental health literacy than non-Indigenous Australians.

A clinical yarning approach [see 294] could help mitigate any potential barriers with establishing therapeutic rapport, service engagement and possible referral pathways. When making recommendations for treatment, health professionals should be aware that Indigenous peoples often face multiple access barriers (e.g., cost, transport, limited range of service for rural and remote communities) especially when needing to access multiple and ongoing health care as is required for eating disorder treatment.

Health professionals are also encouraged to conceptualise eating disorders from the perspective of social emotional wellbeing [see 295]. Social emotional wellbeing is phrase and holistic concept of health unique to Aboriginal and Torres Strait Islander peoples and distinguishes the understanding of mental health disorders from the medical orientated, euro-centric conceptualisation of mental health and treatment. As such, standard nutrition guidance may not be suitable for Aboriginal and Torres Strait Islander People who are accustomed to living off the country or are experiencing high rates of food insecurity [296]. Additionally, it is important for health professionals to understand that the shame experienced by some people with disordered eating behaviours may vary across cultures and a tailored understanding of shame in the context of Aboriginal and Torres Strait Islander people is necessary.

It is also recommended that health professionals practice and provide trauma informed care (see Box 3) due to the ongoing and intergenerational trauma, grief and loss consequential to colonisation and its continual impact on contemporary Aboriginal and Torres Strait Islander peoples [297]. Practicing cultural reflexivity (i.e., critically examining one’s own attitudes, values and biases) is a step towards cultural competency. Working in true partnership with Aboriginal and Torres Strait Islander people (i.e., acknowledging the person experiencing the eating disorder, their family and community as equally experts in the process) and collaborating with Aboriginal-led medical and community services or Aboriginal allied health professionals may foster cultural safety and improve engagement [297].

Finally, health professionals are encouraged to broaden their perspective of what constitutes an Indigenous person’s support system which may often involve input from Elders, community members, extended family and friends. It is also important to explore the role of Traditional Healers and bush medicines people, where and if appropriate.

#### LGBTIQA+ 12individuals

Research on the prevalence of eating disorders in gender and sexual minority people is limited, however, emerging research suggests higher rates of eating disorders in LGBTIQA  (lesbian, gay, bisexual, transgender, gender diverse, intersex, queer, asexual and questioning) people compared to their heterosexual and cisgender peers [298, 299]. Health professionals may need to hold in mind additional considerations and tailor aspects of management and communication when working with LGBTIQA+ people with eating disorder who are of higher weight.

Body image dissatisfaction is a core symptom and stressor for sexual and gender minorities and a significant risk factor for the development of an eating disorder [300]. This is especially true for the transgender population where higher levels of incongruence between biological and assigned sex and gender identity are related to higher levels of body image dissatisfaction [301]. Clinicians should explicitly seek consent to physical examine a person’s body and have an awareness of the potential distress related to physical examinations, especially when gender dysphoria is present.

Practicing trauma-informed care (see Box 3) is of particular importance when working with LGBTIQA+ people as research suggests this population experiences higher rates of adverse events compared to the general population [302–304]. People from sexual and gender minorities may face additional stressors including ‘minority stress’, i.e., identity-based stress experienced by members of disadvantaged social groups, over and above the general life stressors experiences by all members of society [305] as well as ‘intra-minority stress’ i.e., stress derived from within the LGBTIQA+ community [306]. LGBTIQA+ people with eating disorders who are of higher weight endure ‘double stigma’ (i.e., weight stigma as well as the stigma from being in a minority group) and associated prejudice and discrimination [307, 308].

Research suggests that 40% of transgender people with an eating disorder did not disclose their gender identity to their clinicians due to fears (based on past experiences with health professionals) of being ignored, stigmatized and/or discriminated against [309]. It is important that health professionals foster a sense of safety by being gender affirmative and do not make assumptions about a person’s gender or sexual identity. This may include asking the person experiencing an eating disorder about their pronouns and seeking consent before disclosing their gender or sexual identity to other health professionals, family members and/or supports. Using gender neutral language when discussing management with people experiencing eating disorders (e.g., swapping the terms ‘breast’ for the term ‘chest’; ‘motherhood’ for ‘parenthood’; and ‘breastfeeding’ for ‘nursing’) and may help validate a person’s gender identity and foster a safe healthcare environment. Health professionals are encouraged not to make assumptions about people’s body image and/or body image distress as stereotypes of an ‘ideal body/shape/weight’ may vary across LGBTIQA+ cultures.

Finally, clinicians are encouraged to expand their perspective of what constitutes a family and support system to include ‘chosen and created families’ (i.e., non-nuclear supports) who may provide vital support throughout the treatment journey for people with an eating disorder who are of higher weight. While this also applies to both heterosexual and cisgender people, it is of particular importance for LGBTIA+ people, who, when compared to heterosexual cisgender people are more likely to live alone, less likely to have children and more likely to be estranged from their biological family [310].

## Discussion

A summary of key recommendations9 is provided in Table 12.

**Table 12:** Summary of key recommendations

Recommendations for the management of people with eating disorders who are of higher weight	Level of evidence
Management overview	
All treatment should be provided in the context of interprofessional collaborative practice	C
Psychological therapy for adults	
Psychological treatment should be offered as first-line treatment approach for bulimia nervosa or binge-eating disorder (BED)	A
Cognitive behaviour therapy (CBT) for an eating disorder either in standard form or therapist guided self-help should be considered as first-line treatment in adults with bulimia nervosa or BED	B
Other psychological treatments with evidence such as interpersonal psychotherapy (IPT) and dialectical behaviour therapy (DBT) should be considered as second-line treatment options in adults with bulimia nervosa or BED	B
Other feeding or eating disorder (OSFED), unspecified feeding or eating disorder (UFED) or subsyndromal eating disorders should be treated with treatment recommended for the most similar disorder	C
Consider using therapies utilising non-dieting principles and interventions to reduce disordered eating	D
Therapies with demonstrated efficacy for the treatment of anorexia nervosa* in general, that is cognitive behaviour therapy-enhanced (CBT-E), specialist supportive clinical management (SSCM), Maudsley model of anorexia nervosa treatment for adults (MANTRA) and focal psychodynamic therapy (FPT) should be considered as treatment options	D
Psychological therapy for children and adolescents	
Psychological treatment for an eating disorder should be offered as first-line treatment approach	A
Family based treatment should be considered as first-line treatment for children and adolescents with bulimia nervosa and anorexia nervosa*	B
Other psychological treatments with evidence such as adolescent focused therapy (AFT) and CBT for an eating disorder should be considered as second-line treatment options in children and adolescents with anorexia nervosa* (AFT, CBT) or with bulimia nervosa (CBT)	B
Other psychological treatments with evidence such as CBT for an eating disorder should be considered as second-line treatment options in children and adolescents with bulimia nervosa	B
Children and adolescents with higher weight should be offered a first line evidence-based treatment approach for eating disorders as those who do not have higher weight	C
OSFED, UFED or subsyndromal eating disorders should be treated with treatment recommended for the most similar disorder	C
Pharmacotherapy	
Consider using psychotropic medications with evidence in the treatment of eating disorders	B
Monitor for any non-prescribed use of medication in the context of an eating disorder	D
Physical activity	
Physical activity interventions should focus on physical activity for positive physical and mental health benefits and away from exercising for weight or shape change	C
If compulsive exercise is present, referral to an exercise physiologist experienced in working with larger-bodied people and eating disorders populations is desirable	D
Family and other interventions for adults, adolescents and children	
Include families and other carers when indicated for anyone with an eating disorder	B
Family psychoeducation around impacts of body and eating conversations should include modelling body image acceptance, weight stigma and a focus on health in recovery	D
Nutritional and medical management	
Nutritional/medical guidance should minimise language that can reinforce poor self-worth and contribute to worsening eating disorder behaviours	C
Irrespective of body size, addressing malnutrition and poor diet quality is essential	C

NHMRC grades range: A. Body of evidence can be trusted to guide practice e.g., meta-analyses of randomised controlled trials (RCTs) low risk of bias; B. Body of evidence can be trusted to guide practice in most situations (RCTs or other controlled studies, low risk of bias); C. Body of evidence provides some support for recommendation(s) but care should be taken in its application (moderate risk of bias in trails); and D. Body of evidence is weak and recommendation must be applied with caution (high risk of bias in trails). Full criteria in Additional file 1: Appendix C

* In this guideline, the ICD 11 terminology for anorexia nervosa is adopted rather than the DSM-5 criteria. That is, anorexia nervosa (code 6B80) is used as a broad term to include all people at all body weights and without specifying the underweight criterion (sub coded in ICD-11 as 6B80.0, anorexia nervosa with significantly low body weight). See “Background to eating disorders and how they occur” section for more detail.

## Conclusion

In conclusion, this guideline have compiled a series of recommendation for the approach and care of people with eating disorders who have higher body weight. This guideline has been written from the perspective of the adverse effects of weight stigma and the complexity of causes of eating disorders across people of all sizes. The readers are referred to other literature for management of specific medical and other psychological disorders that are often experienced by people with an eating disorder who are living in a larger body.

## Supplementary Information

**Additional file 1.** **Appendices**. Appendix A - D.

**Additional file 2.** **RIGHT Checklist**. RIGHT (Reporting Items for Practice Guidelines in Healthcare) Statement for Practice Guidelines checklist.

**Additional file 3.** **Quality appraisal**. Quality appraisal of systematic reviews and meta-analyses appraised using JBI critical appraisal checklist for systematic reviews and research syntheses.

## Data Availability

Not applicable.

## Abbreviations

ARFID: Avoidant/restrictive food intake disorder
AFT: Adolescent focused therapy
BED: Binge-eating disorder
BMI: Body mass index
BWLI: Behavioural weight loss interventions
CBT: Cognitive behaviour therapy
CBT-E: Cognitive behaviour therapy-enhanced
DBT: Dialectical behaviour therapy
EMDR: Eye movement desensitisation and reprocessing
FBT: Family based treatment
FPT: Focal psychodynamic therapy
gsh: Guided self-help
HAES: Health at every size®
ICP: Interprofessional collaborative practice
IPT: Interpersonal psychotherapy
LCD: Low calorie diet
LGBTIQA+: Lesbian, gay, bisexual, transgender, gender diverse, intersex, queer, asexual and questioning
MANTRA: Maudsley model of anorexia nervosa treatment for adults
NHMRC: National Health and Medical Research Council
NICE: National Institute for Health and Care Excellence
NEDC: National Eating Disorders Collaboration
OSFED: Other feeding or eating disorder
PCOS: Polycystic ovary syndrome
PRISMA: Preferred reporting items for systematic and meta-analysis
RCT: Randomised controlled trial
rTMS: Repetitive transcranial magnetic stimulation
SSCM: Specialist supportive clinical management
UFED: Unspecified feeding or eating disorder
WHO: World Health Organization

## Footnotes

1 Up to the inclusion of bulimia nervosa in DSM-III in 1980 [1], the only eating disorder that was recognised was anorexia nervosa, historically associated with a low BMI.

2 When BMI is used in this guideline it is broadly based on the World Health Organization (WHO) BMI categories for adults over 20 years old, i.e.: BMI < 18.5 is underweight; BMI 18.5–24.9 is adequate weight; BMI ≥ 25 is overweight; and, BMI ≥ 30 is in an obese weight range [26].

3 OSFED includes presentations that do not meet the full criteria for any of the disorders in the feeding and eating disorders diagnostic class including: atypical anorexia nervosa; bulimia nervosa (of low frequency and/or limited duration); binge eating disorder (of low frequency and/or limited duration); purging disorder; and night eating syndrome.

4 There are other syndromes such as orthorexia nervosa, emotional overeating and food addiction which are also disorders of feeding and/or eating. However, they are not considered in the present guideline as they are outside the major intentional diagnostic schemes. Similarly, diabulimia is not a diagnosable eating disorder, but rather a term used to denote insulin misuse as a weight control behaviour and/or compensation for binge eating episodes in people with diabetes and an eating disorder.

5 This section outlines the current status of treatment and outcomes for all eating disorders, including for people who have low weight anorexia nervosa.

6 NESARC-III (i.e. third wave of National Epidemiologic Survey on Alcohol and Related Conditions) is the largest epidemiologic household survey of US adults that assessed eating disorders.

7 Note. Table 4 does not contain a comprehensive list of all instruments that may be used in the assessment of people with eating disorders who are of higher weight, but as stated, we selected those with the most ro bust evidence. There are many instruments (e.g., Emotional Eating Scale for children and adolescents [EES-C; 138]; Eating in the Absence of Hunger Questionnaire for children and adolescents [EAH-C; 139]; Questionnaire of Eating and Weight Patterns—adolescent version [QEWP-A; 140]; Children’s Eating Attitude Test [ChEAT; [141, 142]); and, the Repe titive Eating Questionnaire (Rep (eat)-Q [157])   that m ay also be used in this context.

8 Restriction is a reduction in dietary intake, eating less food than your body requires. Restraint is the intention to restrict.

9 Guided self-help (self-help material with clinician guidance) is distinct from pure self-help (self-help material only).

10 Palavras et al., [201] was not included in the Brennan et al., review (manuscript in preparation) as the primary outcome was weight loss. It did however investigate a broad range of outcome including quality of life, eatin g dis order psychopathology and physical health stature and met the inclusion criteria for the scope of this guideline.

11 It should be noted that in long-term follow-up of interventions aimed to reduce disorders associated with high weight, improvements have been reported in body dissatisfaction and binge eating frequency [254–257], However, these are studies of high weight disorder and not of people with both an eating disorder and with high weight for whom the results cannot be directly applied and are out of scope for this guideline.

12 It is acknowledged that there are differences across issues of gender identity compared with sexual identity, however for ease of reference, both are included in this section. It is also acknowledged that some terminology is contested and language in this area is evolving.
