# Supplementary material for: Management of eating disorders for people with higher weight: clinical practice guideline
Source: J Eat Disord. 2022 Aug 18;10:121. doi: 10.1186/s40337-022-00622-w (PMC9386978; doi:10.1186/s40337-022-00622-w)
Supplement: Supplementary file 1 — Additional file 1. Appendices. Appendix A - D. [file 40337_2022_622_MOESM1_ESM.docx]

Appendix A: Guideline development group author information

Leah Brennan

Associate Professor Leah Brennan is an Associate Professor in Psychology in the School of Psychology and Public Health at Latrobe University. Leah is a clinical, health, educational and developmental psychologist and a board approved supervisor. Her areas of clinical research focus include eating, weight and body image (e.g., eating disorders, overweight/obesity, body image, weight stigma), and the promoting the availability of best-practice psychology treatment (e.g., telehealth psychology, guided self-help). She works as both an academic (teaching and research) and a clinician. Leah has a particular interest in the application of psychological approaches to understand, prevent and treat eating, weight and body image concerns and their biopsychosocial comorbidities (e.g., polycystic ovary syndrome, depression, stigma) in children, adolescents and adults. She leads the Body Image, Eating and Weight Clinical Research Team (BEWT).

Leah is a member of the National Eating Disorders Collaboration Steering Committee and is the inaugural convenor of the Australian Psychological Society's Eating, Weight and Body Image Interest Group. She is passionate about the provision of best-practice psychological treatment in the community, and the development, evaluation and dissemination of evidence-based interventions suitable for ‘real-world’ application. She also has a particular interest in the provision of best-practice psychological support in rural areas (e.g., optimising the use of telehealth psychology to facilitate access to treatment).

Susan Byrne

Dr Susan Byrne is an Honorary Fellow in the School of Psychology, University of Western Australia, and the Clinical Director at The Swan Centre. completed her clinical qualification (M.Psych/PhD) at the University of Western Australia (UWA) in 1998. In 1998, she also won a Wellcome Trust Prize Studentship and an Overseas Research Award to study toward a D.Phil in Clinical Medicine at Oxford University, Department of Psychiatry. In Oxford, Susan joined a leading international research team headed by Prof. Christopher Fairburn (a Wellcome Trust Principal Research Fellow). She was awarded her D.Phil, in September 2001 and returned to UWA in 2002 to take up a UWA Postdoctoral Research Fellowship. In 2003, she was awarded an NHMRC Postdoctoral Fellowship. In 2003, she was also awarded the Tracey Goodall Award for research and clinical innovation that has made an outstanding contribution to cognitive-behaviour therapy in Australia. In 2008, she took up the position of Senior Research Fellow in the School of Psychology at UWA and, in 2009, was promoted to Associate Professor. Susan is also Clinical Director of the recently established Swan Centre - a private, outpatient service specialising in the treatment of eating disorders for children, adolescents and adults

Over the last 10 years, Susan has secured grants with, and published with, other leading Australian and international eating disorder researchers including Profs Christopher Fairburn and Zafra Cooper (Oxford University), Profs Janet Treasure and Ulrike Schmidt (Institute of Psychiatry, London) Dr Virginia McInotsh (Otago University, NZ), Prof. Ross Crosby (Fargo, U.S.A.), Prof. Barry Marshall and Prof. Cindy Bulik. Her research in the field of eating disorders is highly regarded internationally, where the fact that she is both a researcher and a clinician providing treatment to eating disorder sufferers provides a uniquely-informed perspective to her research. Susan has worked to translate her research into health outcomes and policy by (1) using WA research data regarding the prevalence of eating disorders in the community to initiate the formation of the first public outpatient eating disorders service for youth and adults in WA; (2) membership of the steering committee of the National Eating Disorder Collaboration (NEDC), (3) membership of the NMHRC Obesity Guidelines Development Committee which resulted in the 2013 publication of new clinical guidelines for the management of obesity in primary care (4) Member of the Butterfly Foundation Clinical Advisory Group and (5), member of the National Technical Advisory Group on Eating Disorders.

Belinda Caldwell

Belinda Caldwell is the CEO of Eating Disorders Victoria. She is passion about bringing together her skills, passion and life experience to make a lasting positive difference to the eating disorders journey as experienced by those with an eating disorder, those who care for them and those who treat them. Belinda has a Master of Public Health, Graduate Diploma of Applied Science (Midwifery) and a Diploma Applied Science (Nursing). She has previously held positions as a Carer Consultant and Project Manager at the Centre of Excellent in Eating Disorders (CEED), Board Director/Deputy Chair at Eating Disorders Families Australia, Executive Director at Families Empowered and Supporting Treatment of Eating Disorders (EDFA). In addition to her eating disorders specific roles, Belinda has held positions as a Primary Health Care Consultant, Director at Belinda Caldwell Consulting, Chief Executive Officer at the Australian Practice Nurses Association (APNA) and a Population Health Consultant at General Practice Victoria.

Phillipa Hay

Professor Hay, MD DPhil FRANZCP, is Foundation Chair of Mental Health at Western Sydney University (WSU) and Senior Consultant Psychiatrist at Campbelltown and Camden hospitals in South West Sydney. She is committed to research that results in a better understanding of eating and related disorders to reduce the individual, family and community burden. Her current research focuses on randomised controlled trials of interventions for anorexia nervosa and other eating disorders, as well as public health and community interventions that will reduce barriers to accessing care. She led the working group for the Royal Australian and New Zealand College of Psychiatrists national guidelines for eating disorder treatments. Other studies have explored the distribution, determinants, and diagnostic status of disordered eating behaviours, and eating disorder mental health literacy, in community, clinical and professional samples. At WSU, she is academic lead of the multidisciplinary [Eating Disorder and Body Image Network (EDBI).](https://www.westernsydney.edu.au/thri/research/mental_health_and_wellbeing/eating_disorders_and_body_image)

Phillipa has published widely and her work has received natation and international recognition. In 2015 she received the Lifetime Leadership Award from the ANZ Academy for Eating Disorders, and in 2020 she was awarded the RANZCP Senior Research Award. She led the introduction of the first open access journal in her respected research area (*Journal of Eating Disorders*), is its current Editor-in-Chief and also serves on the Editorial Boards of the *International Journal of Eating Disorders* and*Eating Behaviours*. Her work has been supported by the NHMRC, ARC and CAPES research foundation (Brazil) where in 2015 she was awarded a Science Without Borders Visiting Professorship.

Jo Farmer

Jo Farmer has lived with disordered eating, primarily binge-eating disorder, since she was a teenager. Her experiences, positive and negative, of seeking help for an eating disorder as a person living in a larger body has led to her interest in the development of these guidelines. Jo also lives with a number of other mental health conditions. As a lived experience advocate, she has contributed to work with several not-for-profit organisations. She also writes about her experiences online and has provided submissions to several public inquiries on the mental health system.

Professionally, Jo combines her lived experience with policy and evaluation skills, as an independent evaluation consultant in the mental health and family violence sectors. In this capacity, she is particularly interested in developing evaluations in collaboration with people with lived experience. She has conducted evaluations for government departments, PHNs and mental health organisations. Before becoming an independent consultant, Jo previously led evaluation, policy and strategy in the workplace mental health team at Beyond Blue, and worked with leading consulting companies.

Laura Hart

Dr Laura Hart is a Senior Research Fellow at the Centre for Mental Health, University of Melbourne, an honorary Fellow at La Trobe University’s School of Psychology and Public Health and Fellow of the Strategic Training Initiative for the Prevention of Eating Disorders (STRIPED) at Harvard T. H. Chan School of Population Health and Boston Children’s Hospital.

Laura specialises in population mental health research with a focus on developing and evaluating mental health literacy and prevention programs for the public. She has led the development, evaluation and international dissemination of two mental health programs: *Confident Body, Confident Child* (CBCC) a program for parents of 2-6-year-old children to prevent body dissatisfaction and disordered eating, plus *teen Mental Health First Aid* (tMHFA) a training course for adolescents on how to support friends experiencing a mental health problem or mental health crisis.

Laura has been awarded just under $5M in research funding and has published 49 peer-reviewed papers (22 first author), 4 books (2 first author) and has been cited 1332 times. She has a Google Scholar h-index 20 and Scopus h-index 18 placing her in the 90^th^ centile of scholars for her level and discipline (Mazzucchelli et al., 2019). Hart has experienced 3 career disruptions in the last 5 years totalling 1.5 years of work lost.

Laura sits on the editorial boards of the *International Journal of Eating Disorders* and *Mental Health and Prevention*; two leading international journals. Hart also sits on the *National Eating Disorders Collaboration* Steering Committee and founder of the *Victorian Eating Disorders Research Network*. She has presented international and national funded keynotes, and won multiple awards and fellowships for scientific excellence, including the 2019 Society for Mental Health Research/Australian Rotary Health *Research Impact* for her work on teen Mental Health First Aid.

Gabriella Heruc

Dr Gabriella Heruc is an Accredited Practising Dietitian with Honours in Psychology, a Master of Nutrition & Dietetics and a PhD in Medicine, and holds the position of Credentialing Director for the Australia & New Zealand Academy for Eating Disorders. She was the first dietitian invited to join the National Eating Disorders Collaboration Steering Committee in 2017, and with over 20 years clinical experience in mental health and 15 years focused on eating disorder treatment in public and private inpatient, day program and outpatient settings, she provides clinical dietetic insight to the current working group. Over the last 10 years, Gabriella has served on the Executive Committee of the peak professional body, the Australia & New Zealand Academy for Eating Disorders (ANZAED), in the member-elected roles of Executive member, Treasurer, President-Elect, President and now Past-President. In these roles, she engaged and built collaborative relationships with expert researchers and stakeholders at state, national and international levels, and partnered with leading national policymakers, influencers and experts. In this time, she chaired the 2012 Annual Conference, established the now annual Autumn Workshop Series, improved ANZAED’s financial management and strategic planning and served on the Medicare Implementation Committee for the establishment of unprecedented disorder-specific Medicare Items for eating disorder treatment and was instrumental in educating the sector on their use. She also led the development and publication of the first ever treatment principles and clinical practice standards for dietitians and mental health professionals providing eating disorder treatment (Journal of Eating Disorders), which aims to increase capabilities among health professionals to improve outcomes for consumers. These are providing the framework for a national credentialing system currently being developed to standardise eating disorder care across health practitioners.

Sarah Maguire

Dr Sarah Maguire serves as Director of the InsideOut Institute for Eating Disorders, a partnership between University of Sydney and Sydney Local Health District. She is a clinical psychologist, researcher, educator and policy maker with 20 years of experience in the field of eating disorders. She has worked across multiple settings including hospital, community, health policy and research. She has secured over $20 million in funding for eating disorder research and service development in the last 2 years and is author of 50 peer reviewed publications. She is a supervisor of clinical teams and trainees, as well as a specialist clinical trainer and board approved supervisor. Sarah has played a significant role in the improvement of services to people with eating disorders, in particular where there were none. In her role as a senior advisor to the NSW Ministry of Health she leads the NSW Service Plan for People with Eating Disorders. She is an invited member of the Federal Government’s Mental Health Standing Committee for the National Eating Disorders Collaboration (NEDC) and the Federal Medicare Review Taskforce (Eating Disorders Committee).

Milan Piya

Dr Milan Piya is a Senior Lecturer in Diabetes at the School of Medicine, Western Sydney University, and works as a clinical academic endocrinologist at Camden and Campbelltown Hospitals. He is Research Lead for the multidisciplinary publicly funded South Western Sydney Metabolic Rehabilitation and Bariatric Program (SWS MRBP) based in Camden Hospital, as well as Clinical Lead for the Inpatient Diabetes Service at Campbelltown Hospital. A large number of patients in the SWS MRBP have mental health problems and eating disorders including binge-eating disorder. He has recently been working on adopting screening questionnaires to identify this risk in all patients within the service, and also monitoring their risk as they progress through the multidisciplinary program. He completed his Endocrinology training in the UK, and his PhD in obesity and metabolism at the University of Warwick, UK, before moving to Sydney in 2017.

Julia Quin

Julia Quin is a passionate advocate, parent and peer support within the eating disorders community. Her daughter became ill with Atypical Anorexia Nervosa at the age of 16 and with the support of clinicians and peers she was able to take a lead role in supporting her refeeding and recovery. Their journey inspired Julia to become active within the eating disorders community and to support carers in guiding their loved ones to full and lasting recovery. Julia holds a position at Eating Disorders Victoria as a Carer Coach, supporting carers of newly diagnosed adolescents. She also currently volunteers for Eating Disorders Victoria as cofacilitator of the monthly rALLY support group. Julia continues to volunteer in an administrative capacity for IEDFS (International Eating Disorders Family Support), sharing, listening and learning from other international carers supporting their loved ones to recovery. Julia also supports families within her local community who are undertaking family-led refeeding. Julia remains optimistic that full recovery from an eating disorder is possible, with perseverance and support. Julia extensive experience in customer service roles has provided her with the skills to work with a wide variety of people and industries, in order to build rapport, establish specific needs and work collaboratively for satisfactory outcomes and solutions.

Angelique Ralph

Dr Angelique Ralph, BPsych(Hons) DClinPsych, PhD, is the Research Lead at the National Eating Disorders Collaboration (NEDC). She completed her PhD through an NHMRC scholarship at the School of Psychology at the University of Sydney and has held research positions at the Sydney School of Public Health at the University of Sydney and in the School of Women and Children’s Health at the University of New South Wales. She is currently an Adjunct Research Fellow at La Trobe University. She is passionate about research translation, that is, bridging the gap between knowledge and action through making high-quality research accessible and relevant to those who can use it. Angelique is also a clinical psychologist and has held clinical positions at the NSW Statewide Eating Disorder Service and the Children’s Hospital at Westmead. She currently works with people living with eating disorders in private practice and has a particular interest in working with adults with severe and enduring eating disorders, people with higher weight and people with co-occurring chronic conditions.

Sarah Trobe

Dr Sarah Trobe, PhD ClinPsych, is a registered Clinical Psychologist and holds the position of National Manager for the National Eating Disorders Collaboration. This work sits across a number of projects focusing on building the system of care for the prevention and treatment of eating disorders, including workforce development, system improvement, and clinical standards. Clinically, Sarah has worked in both private and public settings with children and adolescents experiencing eating disorders as well as other mental health difficulties, and her work in these areas is supported by further studies in nutrition. Sarah’s recent work at the Victorian Centre of Excellence in Eating Disorders (CEED) focused on service and sector development across regional and metropolitan Victoria, coordinating and contributing to CEED’s education and training program, case consultation, and support for clinicians new to working with people with eating disorders. Concurrently, Sarah worked clinically at the Royal Children’s Hospital Weight Management Service, working with children and adolescents with higher weight, presenting with complex physical, psychological, and social difficulties.

Andrew Wallis

Dr Andrew Wallis is a clinical social worker and family therapist. He has been working with adolescents and their families for more than 20 years. Andrew has postgraduate qualifications in Systemic Family Therapy and has had specialist training in the UK and USA for Family Therapy for Anorexia Nervosa, Multiple Family Therapy and Attachment Based Family Therapy. In 2003 with colleagues at The Children’s Hospital, Westmead, he established the first Family-Based Treatment program in Australia, and Multiple Family Therapy for Anorexia Nervosa in 2014. He has been providing training, consultation and clinical supervision in Australia and New Zealand for more than 10 years, helping to support public and private services to implement family therapy approaches to adolescent problems. Andrew has also been involved in a range of research and academic work with more than 30 peer reviewed papers and book chapters including his PhD research on the impact of Family Based Therapy on family relationships during treatment. Andrew’s first book - A Practical Guide to Family Therapy: Structured Guidelines and Key Skills. IP Communications: Melbourne, Australia was published in 2011. Andrew’s current role is Co Lead of the Sydney Children’s Hospital Eating Disorder Service and Deputy Head of Department of Adolescent Medicine where his more recent focus has been service development, including the first and only tertiary level adolescent eating disorder day program in Australia. Andrew is a member of the SCHN Human Research Ethics Committee, NEDC Steering Committee and the NEDC/ANZAED Credentialing Expert Advisory Group.

AJ Williams-Tchen

AJ Williams-Tchen is of Wiradjuri/Wotjobulak background. He has lived experience in eating disorders, trauma and Stolen Generation. He is the Founder and Director of Girraway Ganyi Consultancy. He is the Lead Cultural Facilitator, Social Worker, Counsellor and Mental Health First Aid Instructor. AJ holds the following qualifications: Master of Social Work, Bachelor of Social Work (Hons), Master of Education, Graduate Diploma of Career Education, Graduate Certificate in Career Counselling, Advanced Diploma in Leadership & Management, Advanced Diploma in Community Services Management, Diploma of Community Services (Youth Work), Diploma of Community Services (Disability Work), Diploma of Community Services (Case Management), Diploma of Community Development, Diploma of Community Services (Juvenile Justice & Statutory Supervision), Diploma of Community Services Coordination, Diploma of Management, Diploma of Business, Diploma of Community Services (Alcohol, Other Drugs & Mental Health), Diploma of Governance, Certificate IV in Indigenous Leadership, Certificate IV in Governance, Certificate IV Small Business Management, Certificate IV in Frontline Management, Certificate IV Indigenous Leadership, Certificate IV Aboriginal & Torres Strait Islander Primary Health Care and Certificate, Certificate in Hospital Nursing.  AJ has been awarded the NSW Mental Health Matters Award 2012, the Victorian Indigenous Leadership Award 2016 and the 2021 Reconciliation HART Award ‘Highly Commended’ for the Girraway Ganyi YouTube.   AJ previously worked at Mental Health First Aid Australia (2010-2016) as the National AMHFA Program Manager, and with equity and diversity roles at Victoria Police, RMIT University, Victoria University & Melbourne Museum.  He has been involved in research projects related to mental health first aid guidelines, eating disorders and smoking cessation.

Appendix B: Additional research evidence

The following is a full list of all the meta-analyses, systematic reviews and identified primary trials not included in a referenced systematic review used to inform this guideline..

Alcaraz-Ibáñez, M., Paterna, A., Sicilia, Á., & Griffiths, M. D. (2020). Morbid exercise behaviour and eating disorders: A meta-analysis. *Journal of Behavioral Addictions, 9*(2), 206-224. https://doi.org/10.1556.2006.2020.00027

Alfonsson, S., Parling, T., & Ghaderi, A. (2015). Group behavioral activation for patients with severe obesity and binge eating disorder: A randomized controlled trial. *Behavior Modification, 39*(2), 270-294. https://doi.org/10.1177/0145445514553093

Alvarez-Jimenez, M., Gonzalez-Blanch, C., Crespo-Facorro, B., Hetrick, S., Rodriguez-Sanchez, J. M., Perez-Iglesias, R., & Luis, J. (2008). Antipsychotic-induced weight gain in chronic and first-episode psychotic disorders. *CNS Drugs, 22*(7), 547-562. https://doi.org/10.2165/00023210-200822070-00002

Barton, B. B., Segger, F., Fischer, K., Obermeier, M., & Musil, R. (2020). Update on weight-gain caused by antipsychotics: A systematic review and meta-analysis. *Expert Opinion on Drug Safety, 19*(3), 295-314. https://doi.org/10.1080/14740338.2020.1713091

Citrome, L., Holt, R. I., Walker, D. J., & Hoffmann, V. P. (2011). Weight gain and changes in metabolic variables following olanzapine treatment in schizophrenia and bipolar disorder. *Clinical Drug Investigation, 31*(7), 455-482. https://doi.org/10.2165/11589060-000000000-00000

Citrome, L., Tsai, J., Mandel, M., Deng, L., Grinnell, T., & Pikalov, A. (2019). Effect of dasotraline on body weight in patients with binge-eating disorder. American Psychiatric Association 2019 Annual Meeting,

Cook, B., Wonderlich, S. A., Mitchell, J., Thompson, R., Sherman, R., & McCallum, K. (2016, 2016-7). Exercise in eating disorders treatment: Systematic review and proposal of guidelines. *Medicine and Science in Sports and Exercise, 48*(7), 1408-1414. https://doi.org/10.1249/MSS.0000000000000912

Da Luz, F., Hay, P., Gibson, A. A., Touyz, S. W., Swinbourne, J. M., Roekenes, J. A., & Sainsbury, A. (2015). Does severe dietary energy restriction increase binge eating in overweight or obese individuals? A systematic review. *Obesity Reviews, 16*(8), 652-665.

Dastan, B., Zanjani, S. A., Adl, A. F., & Habibi, M. (2020, Jul). The effectiveness of dialectical behaviour therapy for treating women with obesity suffering from BED: A feasibility and pilot study. *Clinical Psychologist, 24*(2), 133-142. https://doi.org/10.1111/cp.12197

De Hert, M., Yu, W., Detraux, J., Sweers, K., van Winkel, R., & Correll, C. U. (2012). Body weight and metabolic adverse effects of asenapine, iloperidone, lurasidone and paliperidone in the treatment of schizophrenia and bipolar disorder. *CNS Drugs, 26*(9), 733-759. https://doi.org/10.2165/11634500-000000000-00000

de Zwaan, M., Mitchell, J. E., Crosby, R. D., Mussell, M. P., Raymond, N. C., Specker, S. M., & Seim, H. C. (2005). Short-term cognitive behavioral treatment does not improve outcome of a comprehensive very-low-calorie diet program in obese women with binge eating disorder. *36*, 89-99. https://doi.org/10.1016/S0005-7894%2805%2980057-7

Dugmore, J. A., Winten, C. G., Niven, H. E., & Bauer, J. (2020). Effects of weight-neutral approaches compared with traditional weight-loss approaches on behavioral, physical, and psychological health outcomes: A systematic review and meta-analysis. *Nutrition Reviews, 78*(1), 39-55. https://doi.org/10.1093/nutrit/nuz/020

Eldredge, K. L., Agras, W. S., Arnow, B., Telch, C. F., Bell, S., Castonguay, L., & Marnell, M. (1997). The effects of extending cognitive-behavioral therapy for binge eating disorder among initial treatment nonresponders. *International Journal of Eating Disorders, 21*(4), 347-352. https://doi.org/10.1002/(sici)1098-108x(1997)21:4<347::aid-eat7>3.0.co;2-o

Elkington, T. J., Cassar, S., Nelson, A. R., & Levinger, I. (2017). Psychological responses to acute aerobic, resistance, or combined exercise in healthy and overweight individuals: A systematic review. *Clinical Medicine Insights: Cardiology, 11*, 1-23. https://doi.org/10.1177/1179546817701725

Gorin, A. A., Le Grange, D., & Stone, A. A. (2003). Effectiveness of spouse involvement in cognitive behavioral therapy for binge eating disorder. *33*(4), 421-433. https://doi.org/10.1002/eat.10152

Gorrell, S., Flatt, R. E., Bulik, C. M., & Le Grange, D. (2021). Psychosocial etiology of maladaptive exercise and its role in eating disorders: A systematic review. *International Journal of Eating Disorders, 54*(8), 1358-1376. https://doi.org/10.1002/eat.23524

Gow, M. L., Tee, M. S., Garnett, S. P., Baur, L. A., Aldwell, K., Thomas, S., Lister, N. B., Paxton, S. J., & Jebeile, H. (2020). Pediatric obesity treatment, self‐esteem, and body image: A systematic review with meta‐analysis. *Pediatric obesity, 15*(3), e12600.

Grilo, C. M., & Masheb, R. M. (2005). A randomized controlled comparison of guided self-help cognitive behavioral therapy and behavioral weight loss for binge eating disorder. *Behaviour Research and Therapy, 43*. https://doi.org/10.1016/j.brat.2004.11.010

Grilo, C. M., McElroy, S. L., Hudson, J. I., Tsai, J., Navia, B., Goldman, R., Deng, L., Kent, J., & Loebel, A. (2020a). Efficacy and safety of dasotraline in adults with binge-eating disorder: A randomized, placebo-controlled, fixed-dose clinical trial. *CNS Spectrums, 26*(5), 481-490. https://doi.org/10.1017/S1092852920001406

Grilo, C. M., White, M. A., Masheb, R. M., Ivezaj, V., Morgan, P. T., & Gueorguieva, R. (2020b). Randomized controlled trial testing the effectiveness of adaptive "SMART" stepped-care treatment for adults with binge-eating disorder comorbid with obesity. *American Psychologist, 75*(2), 204-218. https://doi.org/10.1037/amp0000534

Guerdjikova, A. I., McElroy, S. L., Welge, J. A., Nelson, E., Keck, P. E., & Hudson, J. I. (2009). Lamotrigine in the treatment of binge-eating disorder with obesity: A randomized, placebo-controlled monotherapy trial. *International Clinical Psychopharmacology, 24*(3), 150-158. https://doi.org/10.1097/YIC.0b013e328329c7b5

Kvam, S., Kleppe, C. L., Nordhus, I. H., & Hovland, A. (2016). Exercise as a treatment for depression: a meta-analysis. *Journal of affective disorders, 202*, 67-86. https://doi.org/https://doi.org/10.1016/j.jad.2016.03.063

Levine, M. D., Marcus, M. D., & Moulton, P. (1996). Exercise in the treatment of binge eating disorder. *International Journal of Eating Disorders, 19*(2), 171-177. https://doi.org/10.1002/(sici)1098-108x(199603)19:2

Lewer, M., Kosfelder, J., Michalak, J., Schroeder, D., Nasrawi, N., & Vocks, S. (2017). Effects of a cognitive-behavioral exposure-based body image therapy for overweight females with binge eating disorder: A pilot study. *Journal of Eating Disorders, 5*(1), 1-12. https://doi.org/10.1186/s40337-017-0174-y

Lie, S. Ø., Rø, Ø., & Bang, L. (2019). Is bullying and teasing associated with eating disorders? A systematic review and meta‐analysis. *International Journal of Eating Disorders, 52*(5), 497-514. https://doi.org/10.1002/eat.23035

McElroy, S. L., Arnold, L. M., Shapira, N. A., Keck Jr, P. E., Rosenthal, N. R., Karim, M. R., Kamin, M., & Hudson, J. I. (2003). Topiramate in the treatment of binge eating disorder associated with obesity: A randomized, placebo-controlled trial. *American Journal of Psychiatry, 160*(2), 255-261. https://doi.org/10.1176/appi.ajp.160.2.255

McElroy, S. L., Guerdjikova, A., Kotwal, R., Welge, J. A., Nelson, E. B., Lake, K. A., Keck Jr, P. E., & Hudson, J. I. (2007). Atomoxetine in the treatment of binge-eating disorder: A randomized placebo-controlled trial. *The Journal of Clinical Psychiatry, 68*(3), 390. https://doi.org/10.4088/jcp.v68n0306

McElroy, S. L., Guerdjikova, A. I., Mori, N., Blom, T. J., Williams, S., Casuto, L. S., & Keck, P. E. (2015). Armodafinil in binge eating disorder: a randomized, placebo-controlled trial. *International Clinical Psychopharmacology, 30*(4), 209-215. https://doi.org/10.1097/YIC.0000000000000079

McElroy, S. L., Hudson, J. I., Grilo, C. M., Guerdjikova, A. I., Deng, L., Koblan, K. S., Goldman, R., Navia, B., Hopkins, S., & Loebel, A. (2020). Efficacy and safety of dasotraline in adults with binge-eating disorder: A randomized, placebo-controlled, flexible-dose clinical trial. *The Journal of Clinical Psychiatry, 81*(5), 1-13. https://doi.org/10.4088/JCP.19m13068

McIver, S., O'Halloran, P., & McGartland, M. (2009). Yoga as a treatment for binge eating disorder: A preliminary study [Article]. *Complementary Therapies in Medicine, 17*(4), 196-202. https://doi.org/10.1016/j.ctim.2009.05.002

Mercado, D., Robinson, L., Gordon, G., Werthmann, J., Campbell, I. C., & Schmidt, U. (2021, Nov 1). The outcomes of mindfulness-based interventions for Obesity and Binge Eating Disorder: A meta-analysis of randomised controlled trials. *Appetite, 166*, 105464. https://doi.org/10.1016/j.appet.2021.105464

Moustafa, A. F., Quigley, K. M., Wadden, T. A., Berkowitz, R. I., & Chao, A. M. (2021). A systematic review of binge eating, loss of control eating, and weight loss in children and adolescents. *Obesity, 29*(8), 1259-1271.

Palavras, M. A., Hay, P., Filho, C. A., & Claudino, A. (2017, Mar 17). The Efficacy of Psychological Therapies in Reducing Weight and Binge Eating in People with Bulimia Nervosa and Binge Eating Disorder Who Are Overweight or Obese-A Critical Synthesis and Meta-Analyses. *Nutrients, 9*(3). https://doi.org/10.3390/nu9030299

Palavras, M. A., Hay, P., Mannan, H., da Luz, F. Q., Sainsbury, A., Touyz, S., & Claudino, A. M. (2021). Integrated weight loss and cognitive behavioural therapy (CBT) for the treatment of recurrent binge eating and high body mass index: A randomized controlled trial. *Eating and Weight Disorders-Studies on Anorexia, Bulimia and Obesity, 26*(1), 249-262. https://doi.org/10.1007/s40519-020-00846-2

Palavras, M. A., Hay, P., Touyz, S., Sainsbury, A., da Luz, F., Swinbourne, J., Estella, N. M., & Claudino, A. (2015). Comparing cognitive behavioural therapy for eating disorders integrated with behavioural weight loss therapy to cognitive behavioural therapy-enhanced alone in overweight or obese people with bulimia nervosa or binge eating disorder: Study protocol for a randomised controlled trial. *Trials, 16*(1), 1-10. https://doi.org/10.1186/s13063-015-1079-1

Parker, K., & Brennan, L. (2015). Measurement of disordered eating in bariatric surgery candidates: A systematic review of the literature. *Obesity Research & Clinical Practice, 9*(1), 12-25. https://doi.org/10.1016/j.orcp.2014.01.005

Parker, K., O’Brien, P., & Brennan, L. (2014). Measurement of disordered eating following bariatric surgery: A systematic review of the literature. *Obesity Surgery, 24*(6), 945-953. https://doi.org/10.1007/s11695-014-1248-4

Peckmezian, T., & Hay, P. (2017). A systematic review and narrative synthesis of interventions for uncomplicated obesity: weight loss, well-being and impact on eating disorders. *Journal of Eating Disorders, 5*(1), 1-15.

Rahmani, M., Omidi, A., Asemi, Z., & Akbari, H. (2018). The effect of dialectical behaviour therapy on binge eating, difficulties in emotion regulation and BMI in overweight patients with binge-eating disorder: A randomized controlled trial. *Mental Health and Prevention 9*, 13-18. https://doi.org/10.1016/j.mhp.2017.11.002

Ruotsalainen, H., Kyngäs, H., Tammelin, T., & Kääriäinen, M. (2015). Systematic review of physical activity and exercise interventions on body mass indices, subsequent physical activity and psychological symptoms in overweight and obese adolescents. *Journal of Advanced Nursing, 71*(11), 2461-2477. https://doi.org/10.1111/jan.12696

Shaw, K. A., Gennat, H. C., O'Rourke, P., & Mar, C. D. (2006). Exercise for overweight or obesity. *Cochrane Database of Systematic Reviews*(4). https://doi.org/10.1002/14651858.CD003817.pub3

Shelley-Ummenhofer, J., & MacMillan, P. D. (2007). Cognitive-behavioural treatment for women who binge eat [Article]. *Canadian Journal of Dietetic Practice and Research, 68*(3), 139-142. https://doi.org/10.3148/68.3.2007.139

Tam, G., & Yeung, M. P. S. (2018). A systematic review of the long-term effectiveness of work-based lifestyle interventions to tackle overweight and obesity. *Preventive Medicine, 107*, 54-60. https://doi.org/10.1016/j.ypmed.2017.11.011

Ulian, M. D., Aburad, L., Da Silva Oliveira, M. S., Poppe, A. C. M., Sabatini, F., Perez, I., Gualano, B., Benatti, F. B., Pinto, A. J., Roble, O. J., Vessoni, A., De Morais Sato, P., Unsain, R. F., & Baeza Scagliusi, F. (2018). Effects of health at every size® interventions on health-related outcomes of people with overweight and obesity: A systematic review. *Obesity Reviews, 19*(12), 1659-1666. https://doi.org/10.1111/obr.12749

Appendix C: NHMRC categories A-D

| NHMRC levels of evidence-base | | | | |
| --- | --- | --- | --- | --- |
| Grade of recommendation | **A** | **B** | **C** | **D** |
|  | **Excellent** | **Good** | **Satisfactory** | **Poor** |
| Level of evidence-base | One or more level I studies with a low risk of bias or several level II studies with a low risk of bias | One or two level II studies with a low risk of bias or a SR/several level III studies with a low risk of bias | One or two level III studies with a low risk of bias, or level I or II studies with a moderate risk of bias | Level IV studies, or level I to III studies/SRs with a high risk of bias |
| Description of grade of recommendation | Body of evidence can be trusted to guide practice | Body of evidence can be trusted to guide practice in most situations | Body of evidence provides some support for recommendation(s), but care should be taken in its application | Body of evidence is weak, and recommendation must be applied with caution |

***Note.*** Adapted from Tables 1 and 2 of (National Health and Medical Research Council, 2009).

| Designations of levels of evidence | | | | |  |  |
| --- | --- | --- | --- | --- | --- | --- |
| Level of evidence | **I** | **II** | **III-1** | **III-2** | **III-3** | **IV** |
| Intervention | Systematic review of level II studies | Randomised controlled trial | Pseudorandomised controlled trial | A comparative study with concurrent controls ^a^ | A comparative study without concurrent controls ^b^ | Case series with either post-test or pre-test/post-test outcomes |

***Note.*** Adapted from Table 3 (National Health and Medical Research Council, 2009).
^a^ i.e., non-randomised experimental trial, cohort study, case-control study, or interrupted time series with a control group. ^b^ i.e., historical control study, two or more single arm study, interrupted time series without a parallel control group.

Appendix D: Table of screening instruments

| Screening | | | |
| --- | --- | --- | --- |
| Tool | **Format** | **Useful for** | **Considerations for use** |
| Eating Disorder Screen for Primary Care (EDS) | 5-items rated on a yes / no scale, a score of 2 or greater suggests further assessment (note, no to question 1 is a score) | Screening for eating disorders in adults with higher weight. Not diagnostic of an eating disorder however can indicate that further assessment is warranted | The ESP is recommended over the SCOFF as the ESP has greater validity in this population and individual items are deemed more relevant |
| Eating Disorder Screen for Primary Care (ESP; Cotton et al., 2003)  Are you satisfied with your eating patterns? (A ‘no’ counts as a score)  Do you ever eat in secret? (A ‘yes’ to this and all other questions counts as a score)  Does your weight affect the way you feel about yourself?  Have any members of your family suffered with an eating disorder?  Do you currently suffer with or have you ever suffered in the past with an eating disorder? | | | |
| Disordered Eating in Diabetes - Revised (DEPS-R) | 16-items rated on a 6-point Likert scale, higher scores equal greater disordered eating (see below for DEPS-R questions) | Screening for disordered eating / eating disorders in adults and adolescents with type 1 diabetes mellitus. Not diagnostic of an eating disorder however can indicate that further assessment is warranted | The utility of the DEPS-R for people with type 2 diabetes is currently unclear |
| Disordered Eating in Diabetes - Revised (DEPS-R; Wisting et al., 2019)  Losing weight is an important goal to me  I skip meals and/or snacks  Other people have told me that my eating is out of control  When I overeat, I don’t take enough insulin to cover the food  I eat more when I am alone than when I am with others  I feel that it’s difficult to lose weight and control my diabetes at the same time  I avoid checking my blood sugar when I feel like it is out of range  I make myself vomit  I try to keep my blood sugar high so that I will lose weight  I try to eat to the point of spilling ketones in my urine  I feel fat when I take all of my insulin  Other people tell me to take better care of my diabetes  After I overeat, I skip my next insulin dose  I feel that my eating is out of control  I alternate between eating very little and eating huge amounts  I would rather be thin than to have good control of my diabetes | | | |
