# Supplementary material for: Management of eating disorders for people with higher weight: clinical practice guideline
Source: J Eat Disord. 2022 Aug 18;10:121. doi: 10.1186/s40337-022-00622-w (PMC9386978; doi:10.1186/s40337-022-00622-w)
Supplement: Supplementary file 3 — Additional file 3. Quality appraisal. Quality appraisal of systematic reviews and meta-analyses appraised using JBI critical appraisal checklist for systematic reviews and research syntheses. [file 40337_2022_622_MOESM3_ESM.docx]

**Additional file 3: quality appraisal**

| **Author (Year)** | **1. Is the review question clearly and explicitly stated?** | **2. Were the inclusion criteria appropriate for the review question?** | **Was the search strategy appropriate?** | **4. Were the sources and resources used to search for studies adequate?** | **5. Were the criteria for appraising studies appropriate?** | **6. Was critical appraisal conducted by two or more reviewers independently?** | **7. Were there methods to minimise errors in data extraction?** | **8. Were the methods used to combine studies appropriate?** | **9. Was the likelihood of publication bias assessed?** | **10. Were recommend-ations for policy and/or practice supported by the reported data?** | **11. Were there specific directives for new research appropriate?** | **Inclusion/ Exclusion** |
| --- | --- | --- | --- | --- | --- | --- | --- | --- | --- | --- | --- | --- |
| Alcaraz-Ibáñez et al. (2020) | Y | Y | Y | Y | Y | Y | Y | Y | Y | Y | Y | Include |
| Alvarez-Jimenez et al. (2008) | Y | Y | Y | Y | Unclear | Unclear | Y | Y | Unclear | Y | Y | Include |
| Barton et al. (2020) | Y | Y | Y | Y | Unclear | Unclear | Y | Y | Unclear | Y | Y | Include |
| Citrome et al. (2011) | Y | Y | Y | N | Unclear | Unclear | Unclear | Unclear | Unclear | Y | Y | Include |
| Cook et al. (2016) | Y | Y | Y | Y | Unclear | Unclear | Y | Y | Y | Unclear | Y | Include |
| Da Luz et al. (2015) | Y | Y | Y | Y | Y | Y | N | Y | Y | Y | Y | Include |
| De Hert et al. (2012) | Y | Y | Y | Y | Y | Unclear | Y | Y | Y | Y | Y | Include |
| Dugmore et al. (2020) | Y | Y | Y | Y | Y | Unclear | Unclear | Y | N | Y | Y | Include |
| Elkington et al. (2017) | Y | Y | Y | Y | Y | Y | Unclear | Unclear | Unclear | Y | Y | Include |
| Gorrell et al. (2021) | Y | Y | Y | Y | Y | Y | Y | Y | Unclear | Y | Y | Include |
| Gow et al. (2020) | Y | Y | Y | Y | Y | Y | Y | Y | Y | Unclear | Y | Include |
| Kvam et al. (2016) | Y | Y | Y | Y | Y | Y | Y | Y | Y | Y | Y | Include |
| Lie et al. (2019) | Y | Y | Y | Y | Y | Unclear | Unclear | Y | N | Y | Y | Include |
| Mercado et al. (2021) | Y | Y | Y | Y | Y | Y | N | Y | Y | Y | Y | Include |
| Moustafa et al. (2021) | Y | Y | Y | Y | Y | Y | N | Y | Unclear | Unclear | Y | Include |
| Paganini et al. (2018) | Y | Y | Unclear | Unclear | N | N | Unclear | Unclear | Unclear | Unclear | Unclear | Exclude |
| Palavras et al. (2017) | Y | Y | Y | Y | Y | Y | Y | Y | Unclear | Y | Y | Include |
| Parker & Brennan (2014) | Y | Y | Y | Y | Unclear | Unclear | Unclear | Y | Unclear | Y | Y | Include |
| Parker et al. (2015) | Y | Y | Y | Y | Unclear | Unclear | Unclear | Y | Unclear | Y | Y | Include |
| Peckmezian & Hay (2017) | Y | Y | Y | Y | Y | Y | Y | Y | Unclear | Y | Y | Include |
| Ruotsalainen et al. (2015) | Y | Y | Unclear | Y | Y | Y | Y | Y | Y | Y | Y | Include |
| Shaw et al. (2006) | Y | Y | Y | Y | Y | Y | Unclear | Y | Y | Y | Y | Include |
| Tam & Yeung (2018) | Y | Y | Y | Y | Y | Y | Y | Y | Unclear | Y | Y | Include |
| Ulian et al. (2018) | Y | Y | Y | Y | Y | Unclear | Unclear | Y | Unclear | Y | Y | Include |

***Note.*** Y = Yes; N = No

Alcaraz-Ibáñez, M., et al. (2020). Morbid exercise behaviour and eating disorders: A meta-analysis. *Journal of Behavioral Addictions, 9*(2), 206-224. https://doi.org/10.1556.2006.2020.00027

Alvarez-Jimenez, M., et al. (2008). Antipsychotic-induced weight gain in chronic and first-episode psychotic disorders. *CNS Drugs, 22*(7), 547-562. https://doi.org/10.2165/00023210-200822070-00002

Barton, B. B., et al. (2020). Update on weight-gain caused by antipsychotics: A systematic review and meta-analysis. *Expert Opinion on Drug Safety, 19*(3), 295-314. https://doi.org/10.1080/14740338.2020.1713091

Citrome, L., et al. (2011). Weight gain and changes in metabolic variables following olanzapine treatment in schizophrenia and bipolar disorder. *Clinical Drug Investigation, 31*(7), 455-482. https://doi.org/10.2165/11589060-000000000-00000

Cook, B., et al. (2016, 2016-7). Exercise in eating disorders treatment: Systematic review and proposal of guidelines. *Medicine and Science in Sports and Exercise, 48*(7), 1408-1414. https://doi.org/10.1249/MSS.0000000000000912

Da Luz, F., et al. (2015). Does severe dietary energy restriction increase binge eating in overweight or obese individuals? A systematic review. *Obesity Reviews, 16*(8), 652-665.

De Hert, M., et al. (2012). Body weight and metabolic adverse effects of asenapine, iloperidone, lurasidone and paliperidone in the treatment of schizophrenia and bipolar disorder. *CNS Drugs, 26*(9), 733-759. https://doi.org/10.2165/11634500-000000000-00000

Dugmore, J. A., et al. (2020). Effects of weight-neutral approaches compared with traditional weight-loss approaches on behavioral, physical, and psychological health outcomes: A systematic review and meta-analysis. *Nutrition Reviews, 78*(1), 39-55. https://doi.org/10.1093/nutrit/nuz/020

Elkington, T. J., et al. (2017). Psychological responses to acute aerobic, resistance, or combined exercise in healthy and overweight individuals: A systematic review. *Clinical Medicine Insights: Cardiology, 11*, 1-23. https://doi.org/10.1177/1179546817701725

Gorrell, S., et al. (2021). Psychosocial etiology of maladaptive exercise and its role in eating disorders: A systematic review. *International Journal of Eating Disorders, 54*(8), 1358-1376. https://doi.org/10.1002/eat.23524

Gow, M. L., et al. (2020). Pediatric obesity treatment, self‐esteem, and body image: A systematic review with meta‐analysis. *Pediatric Obesity, 15*(3), e12600.

Kvam, S., et al. (2016). Exercise as a treatment for depression: a meta-analysis. *Journal of Affective Disorders, 202*, 67-86. https://doi.org/https://doi.org/10.1016/j.jad.2016.03.063

Lie, S. Ø., et al. (2019). Is bullying and teasing associated with eating disorders? A systematic review and meta‐analysis. *International Journal of Eating Disorders, 52*(5), 497-514. https://doi.org/10.1002/eat.23035

Mercado, D., et al. (2021, Nov 1). The outcomes of mindfulness-based interventions for Obesity and Binge Eating Disorder: A meta-analysis of randomised controlled trials. *Appetite, 166*, 105464. https://doi.org/10.1016/j.appet.2021.105464

Moustafa, A. F., et al. (2021). A systematic review of binge eating, loss of control eating, and weight loss in children and adolescents. *Obesity, 29*(8), 1259-1271.

Paganini, C., et al. (2018). The overlap between binge eating behaviors and polycystic ovarian syndrome: An etiological integrative model. *Current Pharmaceutical Design, 24*(9), 999-1006. https://doi.org/10.2174/1381612824666171204151209

Palavras, M. A., et al. (2017, Mar 17). The Efficacy of Psychological Therapies in Reducing Weight and Binge Eating in People with Bulimia Nervosa and Binge Eating Disorder Who Are Overweight or Obese-A Critical Synthesis and Meta-Analyses. *Nutrients, 9*(3). https://doi.org/10.3390/nu9030299

Parker, K., et al. (2015). Measurement of disordered eating in bariatric surgery candidates: A systematic review of the literature. *Obesity Research & Clinical Practice, 9*(1), 12-25. https://doi.org/10.1016/j.orcp.2014.01.005

Parker, K., et al. (2014). Measurement of disordered eating following bariatric surgery: A systematic review of the literature. *Obesity Surgery, 24*(6), 945-953. https://doi.org/10.1007/s11695-014-1248-4

Peckmezian, T., et al. (2017). A systematic review and narrative synthesis of interventions for uncomplicated obesity: weight loss, well-being and impact on eating disorders. *Journal of eating disorders, 5*(1), 1-15.

Ruotsalainen, H., et al. (2015). Systematic review of physical activity and exercise interventions on body mass indices, subsequent physical activity and psychological symptoms in overweight and obese adolescents. *Journal of Advanced Nursing, 71*(11), 2461-2477. https://doi.org/10.1111/jan.12696

Shaw, K. A., et al. (2006). Exercise for overweight or obesity. *Cochrane Database of Systematic Reviews*(4). https://doi.org/10.1002/14651858.CD003817.pub3

Tam, G., et al. (2018). A systematic review of the long-term effectiveness of work-based lifestyle interventions to tackle overweight and obesity. *Preventive Medicine, 107*, 54-60. https://doi.org/10.1016/j.ypmed.2017.11.011

Ulian, M. D., et al. (2018). Effects of health at every size® interventions on health-related outcomes of people with overweight and obesity: A systematic review. *Obesity Reviews, 19*(12), 1659-1666. https://doi.org/10.1111/obr.12749
